# Stem Rust Resistance in a Geographically Diverse Collection of Spring Wheat Lines Collected from Across Africa

**DOI:** 10.3389/fpls.2016.00973

**Published:** 2016-07-11

**Authors:** Renée Prins, Susanne Dreisigacker, Zakkie Pretorius, Hester van Schalkwyk, Elsabet Wessels, Corneli Smit, Cornel Bender, Davinder Singh, Lesley A. Boyd

**Affiliations:** ^1^CenGen (Pty) Ltd.Worcester, South Africa; ^2^Department of Plant Sciences, University of the Free StateBloemfontein, South Africa; ^3^International Maize and Wheat Improvement CentreMexico City, Mexico; ^4^Faculty of Agriculture and Environment, Plant Breeding Institute Cobbitty, University of SydneyNarellan, NSW, Australia; ^5^Department of Genetics and Breeding, National Institute of Agricultural BotanyCambridge, UK

**Keywords:** adult plant resistance, genome wide association study, hexaploid wheat, *Puccinia graminis* f. sp. *tritici*, *Triticum aestivum*, Ug99

## Abstract

Following the emergence of the Ug99 lineage of *Puccinia graminis* f. sp. *tritici* (*Pgt*) a collective international effort has been undertaken to identify new sources of wheat stem rust resistance effective against these races. Analyses were undertaken in a collection of wheat genotypes gathered from across Africa to identify stem rust resistance effective against the *Pgt* races found in Eastern and Southern Africa. The African wheat collection consisted of historic genotypes collected in Kenya, South Africa, Ethiopia, Sudan, Zambia, Morocco, and Tunisia, and current South African breeding lines. Both Bayesian cluster and principal coordinate analyses placed the wheat lines from Sudan in a distinct group, but indicated a degree of genetic relatedness among the other wheat lines despite originating from countries across Africa. Seedling screens with *Pgt* race PTKST, pedigree information and marker haplotype analysis confirmed the presence of *Sr2, Sr36, Sr24, Sr31*, and *Lr34/Yr18/Sr57* in a number of the lines. A genome-wide association study (GWAS) undertaken with Diversiry Arrays Technology (DArT) and stem rust (*Sr*) gene associated markers and Stem Area Infected (SAI) and Reaction Type (RT) field phenotypes, collected from trials carried out across two seasons in Kenya in 2009 and in South Africa in 2011, identified 29 marker-trait associations (MTA). Three MTA were in common between SAI and RT, with the biggest effect MTA being found on chromosome 6AS. Two wheat lines, W1406 and W6979 that exhibited high levels of adult plant stem rust resistance were selected to generate bi-parental mapping populations. Only the MTA on chromosomes 6AS and 3BS, and the locus *Lr34/Yr18/Sr57* were confirmed following QTL mapping. Additional stem rust resistance QTL, not detected by the GWAS, were found on chromosomes 2BS, 2DL, 3DL, and 4D.

## Introduction

Across Africa wheat consumption has increased considerably since the mid 1990's, faster than any other major food grain. This has resulted in a growing reliance on wheat imports, as wheat production in Africa has failed to keep up with demand (Mason et al., 2012). Wheat is a non-traditional crop in Africa and is believed to have been brought to the continent via several different routes. Historically wheat entered north-western and coastal Eastern Africa from the Mediterranean region via trans-Saharan caravans (Morris and Byerlee, 1993). Durum wheat has been grown in the Abyssinian highlands of Ethiopia, and along the Nile valley in Egypt and the Sudan for thousands of years (Bonjean and Angus, 2001). Bread wheat was introduced into Southern Africa by the Dutch in the Seventeenth Century and by missionaries into East Africa, including Kenya and Tanzania in the Nineteenth Century (Bonjean and Angus, 2001). Wheat yields in Africa have tended to be low, but recent wheat simulation analyses suggest that there may be potential for profitable, competitive, wheat production in several African countries (Shiferaw et al., 2012).

Disease is a major constraint to wheat production across Africa, with the rust pathogens being a significant problem. The wheat growing regions of Eastern Africa are proven hot-spots for the origin of new races of wheat rust, especially stem and stripe rust (Singh et al., 2006). In 1999 a new race (TTKSK) of the stem rust pathogen *Puccinia graminis* f. sp. *tritici* (*Pgt*), commonly known as Ug99, was reported in Uganda (Pretorius et al., 2000). This new race soon spread to the neighboring countries of Kenya and Ethiopia. Ug99 was virulent to the widely deployed stem rust resistance gene *Sr31* that had been effective for many years (Singh et al., 2006). Variants of this race with added virulence to *Sr24* (race TTKST) and *Sr36* (race TTTSK) were subsequently identified in Eastern Africa in 2006 and 2007, respectively. At present 13 variants within the Ug99 race group have been identified from Egypt to South Africa, as well as in Yemen and Iran (http://www.rusttracker.org, accessed 16th February 2016). The Ug99 related race PTKST, with virulence for *Sr31* was detected in South Africa in 2009 where it is believed to be an exotic introduction (Visser et al., 2011). In addition to the Ug99 threat, the non-Ug99 *Pgt* race TKTFF resulted in stem rust epidemics on the cv. Digalu in Ethiopia in 2014 (Olivera et al., 2015).

The appearance of the Ug99 race lineage resulted in an international effort to characterize existing sources and identify new genes for stem rust resistance through the Durable Rust Resistance in Wheat project coordinated by Cornell University (http://www.wheatrust.cornell.edu). Both genome-wide association studies (GWAS) and bi-parental mapping approaches have been used in the genetic identification of new stem rust resistance genes (Yu et al., 2014). GWAS relies on the linkage between genes and molecular markers having been broken by many generations of recombination, with any remaining associations being due to close physical proximity. GWAS therefore overcomes two fundamental limitations of bi-parental mapping: the limited amount of recombination that occurs during the development of the mapping population, which in turn influences the resolution of QTL positions, and the limited allelic diversity that segregates within a bi-parental cross. However, GWAS is prone to false positive QTL identification due to population structure (Korte and Farlow, 2013). Subsequent mapping in bi-parental populations allows the validation of marker-trait associations (MTA), confirming the presence of QTL carrying regions.

Currently, Kielsmeier-Cook et al. (2015) and Singh et al. (2015) list *Sr2, 9h, 13, 14, 15, 21, 22, 24, 25, 26, 27, 28, 29, 32, 33, 35, 36, 37, 39, 40, 42, 43, 44, 45, 46, 47, 50, 51, 52, 53, 55, 57, 58, Huw234, ND643, Yae, SrTA10171, SrTA1662, SrTA10187, SrTmp, and Sr1RS*^Amigo^ as effective to at least one pathotype within the Ug99 race group. In addition, a number of effective stem rust resistance QTL have been identified, Yu et al. (2014) listing 141 loci for resistance to Ug99, including major genes and QTL. However, *Sr9h, Sr21, Sr24, Sr36* and *SrTmp* have failed to individual Ug99 races, while effective to others (Singh et al., 2015; Patpour et al., 2016). Furthermore, not all genes in the above list are exploitable due to inadequate protection levels in adult plants, occurrence of virulence in other Pgt races, or undesirable linkage drag (Singh et al., 2015). Thus, there is still a need to identify new stem rust resistance genes, and in particular sources of wheat APR that may prove more durable.

The objectives of this study were (i) to evaluate a collection of hexaploid spring wheat genotypes collected from across Africa for stem rust resistance effective against the Ug99 lineages, (ii) to determine the genetic diversity and population structure within this African wheat collection, including identifying haplotypes of known *Sr* genes, (iii) to identify genomic regions conferring stem rust resistance via GWAS and (iv) to corroborate the GWAS results in two selected stem rust resistant wheat lines through bi-parental QTL analyses.

## Materials and methods

### Plant materials, field and seedling stem rust resistance tests

A collection of 223 wheat accessions, gathered from across Africa, was obtained from the Genome Resource Unit (GRU), Norwich Research Park, UK (Table 1). Six plants from each accession were grown at New Found Farm, Norwich, to multiply seed and assess homozygosity. Seventy South African advanced breeding lines and commercial wheat varieties were added to the collection. All lines were grown in the 2009 off-season field trials at the Kenyan Agricultural and Livestock Research Organisation (KALRO)-Njoro. Lines were assessed for growth type, homozygosity and stem rust resistance, and two lines of each accession were selected for further greenhouse seed multiplication in South Africa. These lines were subsequently screened in the 2009 main-season field trials at KALRO-Njoro and in field trials near Greytown, South Africa in 2011. The lines were also included in greenhouse seedling tests at the University of Free State, South Africa in 2010. A final panel of 256 African genotypes was identified for the GWAS (Supplementary Table 1).

**Table 1:** **Country of origin of African wheat accessions used in Genome Wide Association Study**.

**Country of origin**	**No. of entries**
Ethiopia	49
Kenya	103
Morocco	8
South Africa: old	6
South Africa: modern	39
Sudan	37
Tunisia	12
Zambia	2

The KALRO-Njoro off- and main-season trials were planted in December 2008 and June 2009, respectively. Entries were hand sown in twin rows 0.7 m long and spaced 0.3 m apart. Rust spreader plants were inoculated with the *Pgt* race TTKST at jointing stage using an ultra low-volume application of a spore suspension in light mineral oil. Plots were fertilized with N:P:K according to recommended rates, hand weeded and irrigated at regular intervals to supplement rainfall. At Greytown the African wheat collection was planted at the Pannar Research Station in May 2011. Entries were hand sown in single 1 m rows spaced 0.7 m apart. The stem rust-susceptible wheat line 37-07 was sown in all pathways bordering plots. Fertilizer (2:3:4 [38] Zn) was applied at a rate of 250 kg/ha at planting, and weeds were chemically and manually controlled. The Ug99-related race PTKST, detected in South Africa in 2009 (Visser et al., 2011), was inoculated onto spreader blocks of line 37-07 (4 rows per block). Fresh urediniospores were suspended in light mineral oil and applied using an ultra low-volume sprayer. Inoculated spreader blocks were allowed to dry before covering overnight with plastic sheeting to create a high-humidity environment.

One score date from the off- (scored March 2009) and main-season (scored September 2009) field trials at Njoro were used in the GWAS. The Greytown trial was scored three times, at weekly intervals from the end of October to the beginning of November 2011: scores A, B, and C. The modified Cobb scale (Peterson et al., 1948) was used to assess stem rust resistance, with percentage severity measured as the stem area infected (SAI) with pustules and host reaction type (RT) measured as resistant (R), moderately resistant (MR), and moderately susceptible (MS) to susceptible (S). Where RTs overlapped, scores such as RMR, MRMS or MSS were recorded. RTs were rescaled from 1 (R) to 7 (S) for statistical analyses.

Two stem rust resistant lines; GRU accession codes W1406 (line Kenya_TK_42; Pedigree: (Penjamo-62/908-Frontana-1)//Kentana-54-B) and W6979 (commercial variety Kenya-Popo; Pedigree: Klein-Atlas/Tobari-66//Centrifen/3/Bluebird/4/Kenya-Fahari), were selected for further study. These lines were crossed with the stem rust susceptible line 37-07 (stem rust susceptible selection from 2007 Stem Rust Trap Nursery, South Africa: Pedigree Kasyob/Genaro-81/Cham4) and doubled haploid (DH) populations generated from F_1_ seed by Sensako (Pty) Ltd using the maize pollination technique (Laurie and Bennett, 1988). Stem rust resistance was assessed in both DH populations, 184 lines per population, in field trials at Pannar Research Station, Greytown in 2012, 2014 and 2015, and at Makhathini Research Station, Jozini, South Africa in 2014. The W1406 x 37-07 population was also tested at Jozini in 2013. All field trials were inoculated with *Pgt* race PTKST. These field trials were scored once within a season at each location using the same stem rust scoring system as used for the African wheat collection.

To identify all-stage stem rust resistance genes within the African wheat collection seedlings were inoculated with *Pgt* race PTKST according to procedures described by Pretorius et al. (2012). Infection types (ITs) were scored 14 days after inoculation using a 0 to 4 scale (McIntosh et al., 1995). Controls used in the stem rust seedling assay were: McNair (*SrMcN*), Morocco (*Sr* unknown), Federation^*^4/Kavkaz (*Sr31*), SrTt1Sr36 (*Sr36*) and Avocet S (*Sr26*).

### Stem rust resistance gene haplotype analysis in the african wheat collection

DNA was extracted from single wheat seedlings using the CTAB extraction method (Doyle and Doyle, 1990). All 256 lines were screened with published markers for 11 stem rust resistance genes using the published PCR protocols (Supplementary Table 2). Fluorescently labeled primers allowed sequence-based fragment detection on an ABI3730*xl* capillary instrument (Applied Biosystems, Foster City, CA, USA) applied at the Central Analytical Facility of Stellenbosch University, South Africa. GeneScan™ 500 LIZ® or GeneScan™ 1200 LIZ® (Applied Biosystems) was used as an internal size standard. Data were analyzed using GeneMapper v4.0 (Thermofischer, formally Applied Biosystems). A stem rust resistance gene was called as present if the marker-allele at all markers associated with the *Sr* gene were the same as the control line. Controls for known stem rust resistance genes and gene complexes included: Cranbrook (*Sr2*), Sr22B (*Sr22*), Palmiet (*Sr2, Sr24*), Avocet S (*Sr26*), Federation^*^4/Kavkaz (*Sr31*), RL5405 (*Sr33*), Mq12/5^*^G2191_Rsr35 (*Sr35*), SrTt1Sr36 (*Sr36*), RL6082 (*Sr39/Lr35*), Kariega (*Lr34/Yr18/Sr57*) and Pavon 76 (*Lr46/Yr29/Sr58*).

### Whole genome profiling, population stratification and linkage disequilibrium analyses

All 256 wheat genotypes were profiled with Diversity Arrays Technology (DArT) markers (Diversity Arrays Technology Pty Ltd, Australia; http://www.diversityarrays.com) using the composite DArT array v2.6, a high-density array enriched for D-genome markers. A total of 3078 polymorphic DArT markers were reported. Missing values were imputed using the MissMDA package, v1.2 (Husson and Josse, 2010; www.r-project.org). Polymorphic markers with a minimum allele frequency (MAF) < 0.05 and a maximum R-squared (*r*^2^) between markers of *r*^2^ = 1 were excluded from the dataset, leaving 2185 polymorphic DArT markers. The Wheat Interpolated Maps v4 (Diversity Arrays Technology Pty Ltd, personal communication) were used to determine the genetic map positions of the DArT markers. Of the 2185 DArT markers 1704 could be assigned a map location.

To correct for possible non-functional correlations between the African wheat collection's population stratification and the stem rust phenotypes, leading to the detection of false-positive MTA (Type I error), population structure was determined using both Bayesian clustering analyses and principal component analyses (PCA). The number of subpopulations was determined by Bayesian clustering analysis using STRUCTURE v.2.2 (Pritchard et al., 2000). An admixture model with correlated allele frequencies was assumed. The program was run with 84 equally distributed DArT markers for *k-values* 1 to 5, with 50,000 burnin iterations followed by 500000 MCMC (Markov Chain Monte Carlo) iterations for accurate parameter estimation. Five independent runs for each *k* were performed. The most probable number of groups was determined by plotting the estimated likelihood values [LnP(D)] obtained by STRUCTURE runs against *k*. In addition, *delta k* (Evanno et al., 2005) was calculated and plotted equally. For the most probable *k*-value the Q-matrix was extracted from STRUCTURE. PCA was performed in the statistical package R with both a subset (84) and all (2185) DArT markers, and with the *Sr* gene associated markers used in the haplotype analysis.

Genome-wide linkage disequilibrium (LD) among markers was estimated by calculating the *r*^2^-values between each marker pair using the software TASSEL v.3.0 (http://www.maizegenetics.net/tassel). LD values were calculated separately for each chromosome and then combined. The *r*^2^-values were plotted against genetic distance and a Loess curve fitted to determine at which distance the curve intercepts the critical *r*^2^ of 0.1. The *Sr* gene associated markers that were not mapped on the DArT Wheat Interpolated Maps v4 were added according to their chromosome allocation. Multiple alleles for *Sr* gene associated SSR and STS loci were considered as individual markers. Rare alleles (MAF < 0.05) for each marker were pooled to create a single rare allele class. Where the pooled rare allele class was still below 5% the allele class was excluded from the LD and GWAS analyses.

### Genome wide association study for stem rust resistance

Different statistical methods were used to calculate *p*-values that defined associations between markers and stem rust infection scores, taking into account population structure.

The underlying equation for the models was:

$$\begin{array}{l} y=X\alpha +Q\beta +Kv+\varepsilon \end{array}$$

Where *y* is the response vector for the phenotypic values, α is the vector of fixed effects related to the DArT marker, β is the vector related to population structure, *v* is the vector of random effects for co-ancestry and ε is the vector of residuals, while *Q* is the relationship matrix and *K* the identity matrix. Seven models, comprising both general linear models (GLM) and mixed linear models (MLM) were selected. The Q-matrix from STRUCTURE and the first 10 significant principal components (PCs) from the PCA using DArT markers, explaining > 40% of the genetic variance, were used as relationship matrices. Two different kinship matrices were also used to correct for any population structure. The first kinship matrix (K1) was calculated in TASSEL, the second matrix (K2) according to Kang et al. (2008). Results were compared to determine the best model. The following models were tested: (i) Q1: GLM with the Q-matrix as correction for population structure, (ii) Q2: GLM with the PCA eigenvectors as correction for population structure, (iii) K2: MLM with the K2-matrix, (iv) Q1K1: MLM with the Q-matrix and K1-matrix, (v) Q1K2: MLM with the Q-matrix and K2-matrix, (vi) Q2K1: MLM with the PCA eigenvectors and K1-matrix, and (vii) Q2K2: MLM with the PCA eigenvectors and K2-matrix. Since the phenotypic data sets contained missing values the average number of genotypes analyzed in each GWAS varied for each growing season. Adjustments were conducted for minor allele frequencies. The different statistical models were used independently for each trait (IT, SAI, RT) and season (1: Njoro 2008-2009 off-season, 2: Njoro 2009 main-season 3: Greytown 2011, scores A, B, and C) using TASSEL v.3.0 and the Emma approach in R. The critical *p*-values for assessing the significance of MTA were calculated based on a false discovery rate (FDR), with an adjusted *p*-value of 0.05.

### Stem rust resistance QTL identification in doubled haploid populations

DNA was isolated from the parents and individual lines of the DH: W1406 × 37-07 and DH: W6979 × 37-07 populations using the CTAB extraction method. Both populations were screened with SSR markers, KASP™ SNPs and DArT markers. The SSR markers were screened on the 3730*xl* Genetic Analyzer as described above, and represented a core set covering all linkage groups (LGs) and targeted putative QTL carrying chromosomes identified in the GWAS analyses. The parental lines were included in a screen using the iSelect 90K Wheat SNP Array (Wang et al., 2014) and the 35K Axiom® Wheat Breeder's Genotyping Array (http://www.cerealsdb.uk.net/cerealgenomics/CerealsDB/axiom_mapped_snps.php;). Informative KASP™ SNPs were identified and typed in the respective DH population using a 63–57°C touchdown PCR protocol (www.lgcgroup.com). Primer sequences were obtained from CerealsDB (http://www.cerealsdb.uk.net/cerealgenomics/CerealsDB; Wilkinson et al., 2012). The W1406 × 37-07 population was screened with the Wheat-*Pst*I (*Taq*I) (2.3_D) and the W6979 × 37-07 population with the Wheat-*Pst*I (*Taq*I) (3.0) DArT arrays (Diversity Arrays Technology P/L, Canberra, Australia), producing usable data sets of 392 and 817 DArT markers, respectively.

LGs were constructed as described in Agenbag et al. (2012). Composite interval mapping (CIM) was performed with Windows QTL Cartographer v2.51 (Wang et al., 2012), using a forward and backward regression model, a window size of 10 cM and a walk speed of 1 cM. One thousand permutations were performed (P = 0.05) with all 10 phenotypic data sets to determine the LOD threshold above which a QTL was considered as significant. Maps were prepared using MapChart v2.1 (Voorrips, 2002).

### Statistical analyses of phenotypic data

Field SAI and RT scores from the African wheat collection were analyzed as box plots using NCSS v.8 to display the range of SAI scores found for each RT class. The boxes represent the inter-quartile range (IQR; mid 50% data values) of SAI and horizontal lines within each box denote the median. The whisker boundaries were determined by multiplying the IQR by a factor of 1.5. Outliers are indicated by dots. ANOVA, comparing the SAI scores across growing seasons/locations, was carried out using NCSS v.8 for the lines within the African wheat collection. ANOVA of the wheat lines within the W1406 × 37-07 and W6979 × 37-07 DH populations was done with Excel in Microsoft Office v. 2013.

## Results

### Assessment of stem rust resistance within the african wheat collection

Stem rust infection established well in all field trials, allowing a clear scoring of adult plant reactions. In all seasons a broad variation in stem rust reactions was observed within the African wheat collection. The ANOVA of SAI across seasons showed significant differences among genotypes and seasons (Table 2). The mean SAI scores for the African wheat collection were 28.0% in the Njoro off-season, (SAI1), 41.6% in the Njoro main-season (SAI2), and ranged from 13.5 to 53.6% in Greytown for the first (SAI3-Score A) to last (SAI3-Score C) score dates. Reaction type (RT) scores showed similar mean values of 5.6 and 5.5 in the Njoro off- and main-seasons (RT1 and RT2), but were lower at Greytown, ranging from 4.3 to 4.7 for the first (RT3-Score A) to last (RT3-Score C) score dates. More pustule formation (higher SAI values) was seen for each RT class at Greytown (Supplementary Figure 1). The differences in stem rust reactions seen between Njoro and Greytown may be due to different, naturally occurring *Pgt* pathotypes being present in Kenya and South Africa, and/or genotype by environment effects.

**Table 2:** **Analyses of variance using stem area infection (SAI) scores across three seasons, off and main seasons in Njoro, Kenya 2008 to 2009 and Greytown 2011**.

**Source of variation**	**Df**	**SS**	***F*-Ratio**
Genotype	254	252362.2	3.31^*^
Season	2	87612.68	145.99^*^
Genotype × Season	339	101724.8	

* *Term significant at alpha = 0.05*.

There was a general, positive trend between the SAI and RT scores, with greater pustule formation being associated with less necrosis and chlorosis (Supplementary Figure 1). Based on the distribution of infection patterns averaged over the three seasons 8.6% of the genotypes were considered highly resistant (SAI < 20, RT = 1, R) and 30.8% moderately resistant (SAI < 60, 1 < RT = 3, RMR to MR). The disease pressure was slightly higher during the main season at Njoro in 2009 (SAI2) and at Greytown in 2011 (SAI3), both seasons showing a higher percentage of susceptible lines, 44.3 and 38.2% (RT = 7, S), respectively, compared to the off-season at Njoro in 2009 (SAI1), where 26.8% of the lines were susceptible.

### Seedling stem rust resistance and haplotype analysis in the african wheat collection

*Pgt* race PTKST is avirulent for the genes *Sr22, Sr26, Sr33, Sr35, Sr36*, and *Sr39*, but virulent on *Sr24* and *Sr31*. Based on the traditional separation of incompatibility and compatibility according to low and high ITs 20% of the entries were homozygous resistant, while a further 5.4% showed a mixture of ITs toward race PTKST (Supplementary Table 1). Four lines from Kenya and one line from South Africa had a seedling score of IT0. From their pedigrees and the haplotype analyses using *Sr* gene associated markers, the lines were confirmed to carry *Sr36*. The gene *Sr24* was common in current day South African breeder's lines and commercial varieties, being present in 23 of the 45 South African genotypes. Markers also detected *Sr31* in two Kenyan lines and one South African line as was expected based on the presence of “Kavkaz” in their pedigrees. The IT score of all three lines was IT4, equal to the *Sr31* carrying control Federation^*^4/Kavkaz.

The marker haplotype analysis predicted the presence of *Sr35* in a number of lines, but this was not supported by a low IT score (IT; or 0;), or pedigree history, suggesting that the associated markers were not diagnostic for this gene. Markers also predicted the presence of *Sr33* in one Kenyan and six South African lines, but again this prediction was not supported by the expected IT score of IT;1 or 2 with isolate PTKST, or the available pedigree information. Marker analyses did not identify the resistance genes *Sr22, Sr26* or *Sr39* in the African wheat collection. The seedling ITs of the controls were: McNair IT4 (*SrMcN*), Morocco IT4 (*Sr* unknown), Federation^*^4/Kavkaz IT4 (*Sr31*), SrTt1 IT0 (*Sr36*), and Avocet S IT1 (*Sr26*).

Haplotype analysis did indicate the presence of the complex rust APR locus *Lr34/Yr18/Sr57* in 69 of the lines, being particularly common in lines from Kenya and the modern South African lines. However, 10 of the 49 Ethiopian lines described as Ethiopian landraces also contained *Lr34/Yr18/Sr57*. Marker haplotype analysis predicted the presence of *Sr2* in eight Kenyan lines and in two older South African genotypes. The rust APR locus *Lr46/Yr29*/*Sr58* was not called in the African wheat collection using our stringent requirement that all published linked markers must have the same allele as the control genotype carrying *Lr46/Yr29*/*Sr58*, i.e., Pavon 76.

### Genetic population structure and linkage disequilibrium (LD) within the african wheat collection

**Figure 1:**
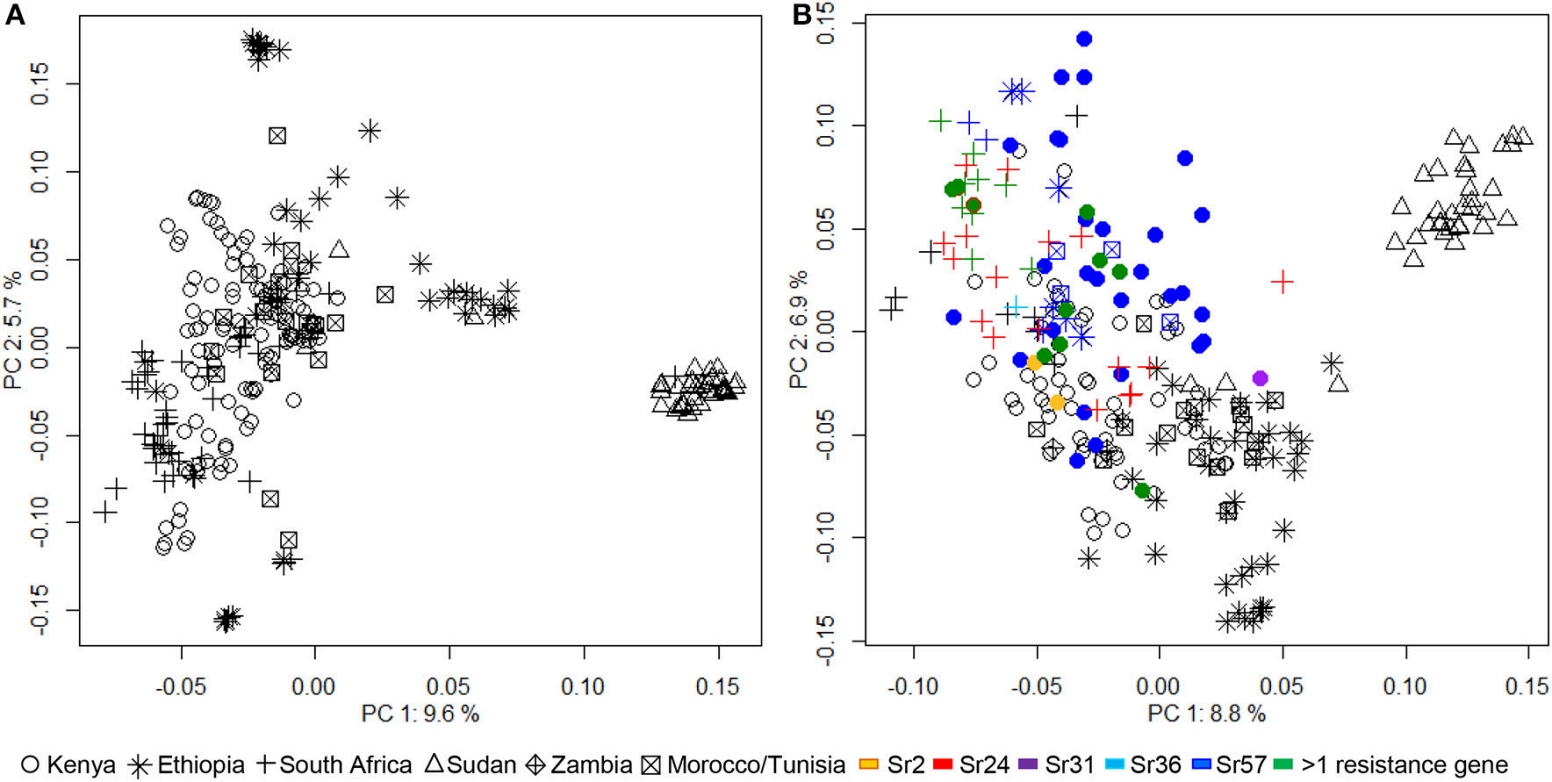
**Analysis of population structure within the African wheat collection calculated by principal component analyses (PCA) performed with (A) DArT markers and (B) 123 simple sequence repeat or sequence tagged sites casual or linked to known ***Sr*** genes**. Lines predicted by haplotype analysis to contain known *Sr* genes *Sr2, Sr24, Sr31, Sr36, Sr57*, or a combination of 2 or more *Sr* genes are indicated.

Bayesian clustering analyses divided the African wheat collection into two subpopulations (Supplementary Figure 2). The first subpopulation contained all the lines from Sudan, while the second included the remaining genotypes. PCA was performed using both DArT and *Sr* gene associated markers, in separate analyses (Figure 1). PCA outputs using a subset of, or all the DArT markers were very similar. The variance described by principal components (PC) was relatively small. PC1 explained 9.6 and 8.8%, and PC2 5.7 and 6.9% of the genetic variance using DArT and *Sr* gene associated markers, respectively. Both PCA plots again placed the Sudanese genotypes in a distinct group, removed from the rest of the collection. The PCA indicated that considerable genetic variance was still tied-up within the remaining genotypes from Kenya, South Africa, Ethiopia, Zambia, Tunisia, and Morocco. PC1 showed a degree of overlap between the Kenyan and South African wheat genotypes which PC2 began to separate into distinct groups. The Ethiopian wheat genotypes generally formed a distinct group, although some lines overlapped with the Kenyan and South African cluster. The lines from Zambia, Morocco, and Tunisia were not distinguishable as separate groups, tending to cluster with the main body of wheat lines from Kenya and South Africa.

LD statistics (*r*^2^, *p*-values) were calculated for each pair of intra-chromosomal DArT and *Sr* gene associated markers. Across the entire data set, only 7.7% of the intra-chromosomal marker pairs showed significant levels of LD (*r*^2^> 0.1, *p* < 0.001). Consequently a low genome-wide intra-chromosomal LD, with an average *r*^2^ of 0.277 was found. A decay in LD with increasing genetic distance was observed, with LD decaying below a critical level (*r*^2^ = 0.1) within a map distance of 5 cM (Supplementary Figure 3). However, the incorrect assignment of DArT markers to chromosome 7DL resulted in a cluster of unmapped marker pairs in the LD decay plot (Supplementary Figure 3).

### Genome wide association study of stem rust resistance within the african wheat collection

Seven different statistical models were used to test for MTA using the seedling IT, field SAI and RT scores. Comparison of cumulative and observed *p*-values for each model indicated larger probabilities of Type 1 error (false positive MTA) when using the STRUCTURE based Q-matrix (Q1) for correction of population stratification (Supplementary Figure 4). We therefore only considered models using the PCA based Q-matrix (Q2) and kinship (K1, K2) to correct for population structure. A stem rust resistance MTA was considered reliable when the association was observed using the MLM, or when the MTA was observed in at least two seasons using the GLM after FDR correction. As expected, the number of significant MTA was higher with the GLM than with the MLM.

The seedling IT scores only identified significant MTA on chromosome 2BS, with all markers displaying a significant association across all models and spanning a 23 cM region (Supplementary Figure 5). The most significant marker in this region was the marker allele *stm773-2-153bp* which is associated with *Sr36* (Tsilo et al., 2008). The DArT markers with the highest *p*-values were *wPt-741721, wPt-744324* and *wPt-6144*, spanning a 0.8 cM region. The two latter DArT markers were in significant LD with the *stm773-2-153bp* marker allele, having *r*^2^-values of 0.170 and 0.649 (*p* < 0.0001), respectively. These MTA were in agreement with the haplotype analysis, stem rust seedling ITs and the pedigree data. Lines predicted to carry *Sr36* were subsequently removed from the African wheat collection before undertaking the GWAS of the field SAI and RT score data sets.

The GWAS of the adult plant SAI scores found significant MTA with two *Sr* gene associated marker alleles, *cssrf6-647bp* and *ncw1-417bp*, and 20 DArT markers (Table 3). The gene associated marker allele *cssrf6-647bp* is positively linked to the rust APR gene *Lr34/Yr18/Sr57* (chromosome 7DS), while the marker allele *ncw1-417bp* is used to follow the APR gene *Lr46/Yr29*/*Sr58* (chromosome 1BL) in informative crosses. The most significant MTA was on chromosome 6AS; DArT marker *wPt-669271* showing associations with SAI2 and all three SAI3 scores. Four additional DArT markers were closely linked to *wPt-669271*, lying within a 2.9 cM region (Table 3). On chromosome 7BL marker *wPt-5377* was significant across all three seasons (SAI1, 2 and 3), although the linked marker *wPt-2356* (distance of 6.6 cM) was only significant in SAI3. Markers *wPt-6487* and *wPt-4842* on chromosome 3BS were significant for SAI1 with all models. Markers on chromosome 2DS (*wPt-730744)*, 3BL (*wPt-0896*) and 6AL (*wPt-734345*) were significant with SAI3-Score B for at least two models. Additional MTA were observed on chromosomes 1BS, 5AS, 6AS, 6BS, and 7DS, markers being significant in one of the two seasons in Njoro and for one to three scores in Greytown 2011. The marker *wPt-667538* was significant for the SAI2 and SAI3 data sets. This DArT marker is located on chromosome 7A (SD, unpublished data), but the genetic position is not known.

**Table 3:** **Markers significantly associated with stem area infection (SAI) phenotypes**.

**Marker**	**Chr. arm**	**Putative position (cM)**	**Stem area infected**^a^																				
			**SAI1**^b^					**SAI2**			**SAI3- Score A**				**SAI3- Score B**					**SAI3- Score C**			
			***K2***	***Q2K2***	***Q2K1***	***Q2***	***r*^2^^c^**	***K2***	***Q2***	***r*^2^**	***K2***	***Q2K2***	***Q2***	***r*^2^**	***K2***	***Q2K2***	***Q2K1***	***Q2***	***r*^2^**	***Q2K2***	***Q2K1***	***Q2***	***r*^2^**
*wPt-9524*	1BS	2.8				1.6E-4	0.05						2.2E-3	0.03				1.5E-4	0.05			2.7E-4	0.05
*wPt-741749*	1BS	34.8				2.3E-4	0.05											2.9E-4	0.05			1.4E-3	0.04
*wPt-1248*	1BS	43.7						7.0E-4	5.7E-6	0.11			7.1E-4	0.04				9.7E-5	0.05			5.0E-5	0.06
*ncw1-417bp*	1BL	115							3.3E-4	0.09			1.9E-4	0.06				1.1E-5	0.08			3.2E-6	0.09
*wPt-730744*	2DS	73.0													4.1E-4			5.1E-4	0.04				
*wPt-6487*	3BS	33.8	8.7E-5	7.4E-4	9.8E-4	3.0E-5	0.06																
*wPt-4842*	3BS	33.8	3.9E-5	6.6E-4	6.9E-4	1.4E-5	0.07																
*wPt-0896*	3BL	84.5											1.1E-5	0.07	2.7E-4			3.7E-8	0.10			2.6E-6	0.08
*wPt-5588*	5AS	40.2				7.7E-5	0.06											8.6E-4	0.04			2.9E-4	0.05
*wPt-669498*	6AS	3.9				1.8E-4	0.05						6.5E-4	0.04				2.2E-5	0.06			2.7E-5	0.06
*wPt-669271*	6AS	5.3							2.4E-6	0.12		9.8E-04	1.5E-7	0.10	3.6E-4	1.1E-4	1.6E-4	1.3E-10	0.14	6.0E-4	5.8E-4	1.7E-9	0.12
*wPt-1742*	6AS	6.3							1.5E-4	0.08			1.3E-5	0.07				2.3E-8	0.11			4.6E-7	0.09
*wPt-666927*	6AS	6.8							3.1E-4	0.07			5.4E-6	0.07				8.5E-8	0.10			4.9E-6	0.07
*wPt-664589*	6AS	6.8																		1.8E-4		1.1E-5	0.07
*wPt-667405*	6AS	23.3				2.7E-4	0.05											2.0E-3	0.03				
*wPt-734345*	6AL	96.3														2.4E-4	2.3E-4						
*wPt-743231*	6BS	62.1				2.5E-4	0.05															9.0E-4	0.04
*wPt-2356*	7BL	210.9														5.9E-4	3.0E-4	3.1E-3	0.03				
*wPt-5377*	7BL	217.5				5.1E-7	0.09		1.4E-5	0.10			5.4E-4	0.04				2.0E-3	0.03			3.5E-4	0.05
*wPt-743854*	7DS	1.1									3.1E-4		7.6E-8	0.10				5.7E-8	0.10			8.0E-6	0.07
*cssrf6-647bp*^d^	7DS	50							4.5E-4	0.08			3.2E-6	0.09				4.4E-6	0.09			9.5E-5	0.06
*wPt-667538*	7A	-							1.3E-4	0.08			8.8E-4	0.04				6.8E-4	0.04				

a *Field infection with UG99 lineage race PTKST*.

b *SAI1: stem area infection, off-season Njoro, Kenya 2009, SAI2: stem area infection, main-season, Njoro, Kenya 2009, SAI3_Score A, B and C: first, second and third score stem area infection, Greytown, South Africa 2011*.

c *r^2^ of model Q2 only*.

d *417bp and 647bp refer to allele classes at marker ncw1 and cssrf6 respectively; Q2 GLM with the PCA eigenvectors as correction for population structure; K2: MLM with the K2-matrix; Q2K1: MLM with the PCA eigenvectors and K1-matrix; Q2K2: MLM with the PCA eigenvectors and K2-matrix*.

For the RT scores the most significant marker association was with *wPt-669271* on chromosome 6AS, identified with RT3 scores and all models, except MLM-K2 (Table 4). This MTA was also found with the SAI phenotypes. Marker *wPt-743231* on chromosome 6BS and marker *wPt-0896* on chromosome 3BL were also significant with both SAI and RT scores in the same seasons (Tables 3, 4). MTA not in common between the SAI and RT GWAS, but in close proximity, were *wPt-6716, wPt-5572, wPt-6995*, and *wPt-731120* on chromosome 6AL, being 2.4 cM distal from marker *wPt-734345* associated with SAI. Similarly, the RT significant marker *wPt-741331* was located 7.3 cM distal from SAI MTA with markers *wPt-6487 and wPt-4842* on chromosome 3BS. For both the 6AL and 3BS MTA markers were significant with the RT1 and RT3 scores, while for SAI the markers were only significant with SAI1. Additional MTA were found on chromosome 2AS with RT1 and RT3-Score B, at two locations on chromosome 2BL with RT2, RT3-Scores B, and C, and on chromosome 6DS with RT1, RT3-Scores B, and C (Table 4).

**Table 4:** **Markers significantly associated with host reaction type (RT) phenotypes**.

**Marker**	**Chr. arm**	**Putative position (cM)**	**Reaction type**^a^																							
			**RT1**^b^					**RT2**				**RT3-Score A**					**RT3-Score B**					**RT3-Score C**				
			***K2***	***Q2K2***	***Q2K1***	***Q2***	***r*^2^^c^**	***K2***	***Q2K2***	***Q2***	***r*^2^**	***K2***	***Q2K2***	***Q2K1***	***Q2***	***r*^2^**	***K2***	***Q2K2***	***Q2K1***	***Q2***	***r*^2^**	***K2***	***Q2K2***	***Q2K1***	***Q2***	***r*^2^**
*wPt-7187*	2AS	20.5				1.0E-4	0.06													1.0E-3	0.04					
*wPt-743061*	2AS	20.5				3.0E-4	0.06													6.0E-4	0.04					
*wPt-2430*	2BL	74.9															8.4E-4			2.6E-5	0.06	4.0E-4			9.9E-6	0.07
*wPt-666931*	2BL	102.1						6.7E-4	5.4E-4	5.2E-6	0.10									2.3E-4	0.05				1.4E-4	0.05
*wPt-741331*	3BS	26.5	1.3E-4			1.6E-5	0.08													3.2E-3	0.03					
*wPt-0544*	3BL	64.1										8.9E-4			3.0E-5	0.07				2.1E-4	0.05				5.2E-4	0.05
*wPt-0896*	3BL	84.5													1.3E-3	0.04	1.4E-4			2.2E-8	0.11				1.9E-8	0.11
*wPt-742982*	3BL	131.4				4.7E-4	0.05													1.7E-4	0.05				6.4E-4	0.04
*wPt-669271*	6AS	5.3										1.7E-4	1.9E-4	1.1E-4	1.8E-9	0.13	7.5E-4	6.6E-4	5.0E-4	7.8E-9	0.12		5.4E-4	3.9E-4	2.2E-10	0.14
*wtPt-6716*	6AL	93.1				3.7E-5	0.07													2.3E-5	0.07				6.0E-5	0.06
*wPt-5572*	6AL	93.9				3.1E-5	0.07													2.8E-5	0.06				1.5E-5	0.07
*wPt-6995*	6AL	93.9				2.4E-5	0.08								8E-4	0.04				6.9E-5	0.06				4.2E-5	0.06
*wPt-731120*	6AL	93.9				5.2E-5	0.07													2.3E-5	0.07				7.5E-6	0.07
*wPt-743231*	6BS	62.1				6.0E-5	0.07													9.8E-4	0.04				7.9E-4	0.04
*wPt-7394*	6DS	21.9	6.3E-4	7.2E-4	3.8E-4	2.2E-5	0.08													4.0E-4	0.05				6.5E-4	0.04

a *Field infection with UG99 lineage race PTKST*.

b *RT1: reaction type, off-season Njoro, Kenya 2009, RT2: reaction type, main-season, Njoro, Kenya 2009, RT3_Score A, B and C: first, second and third reaction type score, Greytown, South Africa 2011*.

c *r^2^ of model Q2 only; Q2 GLM with the PCA eigenvectors as correction for population structure; K2: MLM with the K2-matrix; Q2K1: MLM with the PCA eigenvectors and K1-matrix; Q2K2: MLM with the PCA eigenvectors and K2-matrix*.

### QTL analysis of stem rust resistance in selected wheat lines W1406 and W6979

W1406 and W6979 were selected for bi-parental QTL mapping based on the stem rust resistance scores seen in the field trials in Njoro, Kenya (off-season 2009 and main-season 2009) and Greytown, South Africa (2011) (W1406-0R; 5R; 10R, and W6979-0R; 5R; 40RMR, respectively). The predicted stem rust resistance MTA identified by the GWAS were also considered when selecting lines for bi-parental QTL mapping, thereby providing a wide range of possible stem rust resistance genes for further study (Table 5). W1406 was predicted to carry SAI MTA on chromosomes 2DS, 3BS, 3BL, 5AS, 6BS, 7A, 7BL, and 7DS, and the 7DS locus *Lr34/Yr18/Sr57*. Using the RT phenotypes W1406 was predicted to carry MTA on 2AS, 2BL, 3BS, 3BL, 6AL, 6BS, and 6DS. W6979 was predicted to carry stem rust resistance MTA for SAI on chromosomes 1BS, 3BS, 3BL, 6AS, 7A, 7BL, and 7DS, and *Lr34/Yr18/Sr57*, and MTA for RT on chromosomes 2AS, 2BL, 3BS, 3BL, 6AS, 6AL, and 6DS. Neither W1406 nor W6979 carried *Sr* seedling resistance genes effective against race PTKST (Supplementary Table 1).

**Table 5:** **Stem rust resistance MTA found in wheat lines W1406 and W6979**.

**Marker**	**^a^Chr**.	**Position**	**^b^^,^^d^SAI**	**^c^^,^^d^RT**	**W1406**	**W6979**
*wPt-9524*	1BS	2.8	MTA	None	−	+
*wPt-741749*	1BS	34.8	MTA	None	−	+
*wPt-1248*	1BS	43.7	MTA	None	−	+
*wPt-7187*	2AS	20.5	none	MTA	+	+
*wPt-743061*	2AS	20.5	none	MTA	+	+
*wPt-666931*	2BL	102.1	none	MTA	+	+
*wPt-730744*	2DS	73.0	MTA	None	+	−
*wPt-741331*	3BS	26.5	none	MTA	+	+
*tPt-6487*	3BS	33.8	MTA	None	+	+
*wPt-4842*	3BS	33.8	MTA	None	+	+
*wPt-0544*	3BL	64.1	none	MTA	+	+
*wPt-0896*	3BL	84.5	MTA	MTA	+	+
*wPt-5588*	5AS	40.2	MTA	None	+	−
*wPt-669498*	6AS	3.9	MTA	None	−	+
*wPt-669271*	6AS	5.3	MTA	MTA	−	+
*wPt-1742*	6AS	6.3	MTA	None	−	+
*wPt-666927*	6AS	6.8	MTA	None	−	+
*wPt-664589*	6AS	6.8	MTA	None	−	+
*wPt-667405*	6AS	23.3	MTA	None	−	+
*tPt-6716*	6AL	93.1	none	MTA	+	+
*wPt-5572*	6AL	93.9	none	MTA	−	+
*wPt-6995*	6AL	93.9	none	MTA	−	+
*wPt-731120*	6AL	93.9	none	MTA	−	+
*wPt-743231*	6BS	62.1	MTA	MTA	+	−
*wPt-7394*	6DS	21.9	none	MTA	+	+
*wPt-667538*	7A	unknown	MTA	None	+	+
*wPt-2356*	7BL	210.9	MTA	None	+	+
*wPt-5377*	7BL	217.5	MTA	None	−	+
*wPt-743854*	7DS	1.1	MTA	None	+	+
*cssrf6647*	7DS	50.0	MTA	None	+	+

a *Chr.- chromosome*.

b *SAI- Stem Area Infection*.

c *RT- host Reaction Type*.

d *MTA- Marker Trait Association between phenotype (SAI or RT) and marker. + indicates that the wheat line carries the allele associated with the MTA, while – indicates that is does not*.

The stem rust resistance phenotypes of the parental lines W1406 and W6979 ranged from trace R to 15MR, and trace R to 30MR, respectively, over seasons and locations during field testing of the DH populations in South Africa. The susceptible parent, 37-07 consistently scored 70S to 100S. The W1406 × 37-07 population was field tested over four seasons and two locations, with an ANOVA indicating significant differences in stem rust infection between the DH lines, but not between the five field trials (Supplementary Table 3; Supplementary Figure 6A and 6C). The W6979 x 37-07 population was tested for stem rust resistance over three seasons and two locations. Again significant differences in stem rust reaction were seen between the DH lines, but with this cross significant differences were also seen between the four field trials (Supplementary Table 3; Supplementary Figures 6B, 6D). This may be due to different, naturally occurring *Pgt* pathotypes being present in each season/location to which W6979 showed response variation, and/or genotype by environment effects exhibited by W6979.

**Figure 2:**
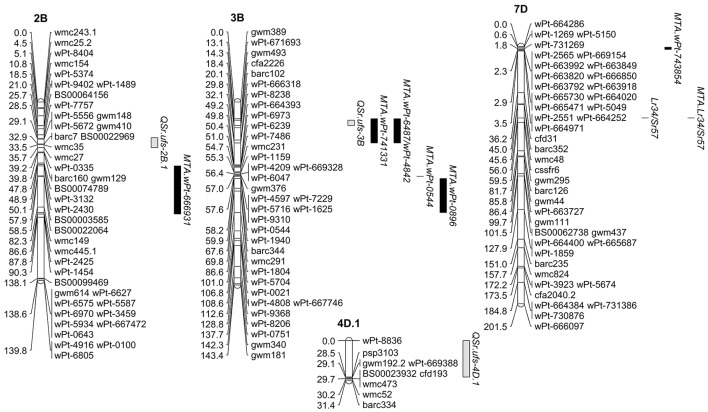
**Stem rust resistance detected in the wheat cross W1406 x 37-07**. The three QTL and *Lr34/Yr18/Sr57* found in the line W1406 are shown along with the MTA detected on the same linkage groups in the GWAS. The complete genetic map generated for the cross W1406 x 37-07 can be found in Supplementary Figure 7.

Genetic maps were produced for both populations incorporating DArT, SSR, and KASP™ SNP markers. The W1406 x 37-07 map covered a genetic distance of 2214.2 cM and incorporated 531 markers (Supplementary Figure 7), while the map for W6979 x 37-07 covered 2667.5 cM and incorporated 859 markers (Supplementary Figure 8). QTL analysis of the W1406 x 37-07 population identified QTL for stem rust resistance on chromosomes 2BS, 3BS, 4A, and 4D, and the rust resistance locus *Lr34/Yr18/Sr57* on chromosome 7DS (Table 6, Figure 2). The most effective QTL in W1406, explaining the greater portion of the genetic variance for stem rust resistance, were the *Lr34/Yr18/Sr57* locus (*r*^2^ up to 22.74% for SAI and 7.15% for RT) and the QTL on 4D, *QSr.ufs-4D* (*r*^2^ up to 26.99% for SAI and 22.68% for RT). *QSr.ufs-2B.1* and *QSr.ufs-3B* were identified in W1406 with only one RT score data set, each contributing a minor effect (*r*^2^ < 10%). A minor QTL was also found on chromosome 4A, *QSr.ufs-4A*, contributed by the susceptible parent, 37-07, but identified with only one RT data set.

**Table 6:** **QTL detected in the bi-parental DH populations of W1406 and W6979 crossed to 37-07 for stem area infected (SAI) and host reaction type (RT) field scores**.

**QTL name^a^**	**Closest marker**	**Chr^b^**		**SAI5 Nov 12**	**RT5 Nov 12**	**SAI31 Jul 13**	**RT31 Jul 13**	**SAI11 Aug 14**	**RT11 Aug 14**	**SAI3 Nov 14**	**RT3 Nov 14**	**SAI15 Oct 15**	**RT15 Oct 15**	**Origin**
**W1406 x 37-07**														
*Lr34/Yr18/Sr57*	*cssrf6*	7D	LOD %VAR^c^	NS^d^	NS	11.86 22.74	NS	NS	3.85 7.15	10.06 15.97	NS	7.04 13.32	NS	W1406
*QSr.ufs-4D*	*wPt-8886 - psp3103^e^*	4D.1	LOD %VAR	NS	6.48 14.2	NS	NS	9.61 26.99	8.71 22.68	NS	NS	6.80 16.44	NS	W1406
*QSr.ufs-2B.1*	*barc7*	2B	LOD %VAR	NS	NS	NS	NS	NS	4.79 8.76	NS	NS	NS	NS	W1406
*QSr.ufs-3B*	*cfa2226*	3B	LOD %VAR	NS	NS	NS	NS	NS	NS	NS	NS	NS	3.24 6.97	W1406
*QSr.ufs-4A*	*wPt667130*	4A	LOD %VAR	NS	3.01 7.67	NS	NS	NS	NS	NS	NS	NS	NS	37-07
**W6979 x 37-07**														
*Lr34/Yr18/Sr57*	*wMAS000003*	7D.1	LOD %VAR	NS	NS	NT^f^	NT	NS	NS	4.01 8.20	NS	6.22 11.02	NS	W6979
*QSr.ufs-6A*	*wPt-4270*	6A	LOD %VAR	5.78 12.39	4.86 9.85	NT	NT	8.34 16.32	7.13 14.57	4.97 10.12	8.49 16.54	7.87 14.10	8.79 17.02	W6979
*QSr.ufs-2B.2*	*gwm148*	2B	LOD %VAR	NS	NS	NT	NT	3.41 6.33	NS	NS	NS	NS	5.44 10.07	W6979
*QSr.ufs-2D*	*wPt-731336*	2D.1	LOD %VAR	NS	4.67 9.58	NT	NT	NS	NS	NS	NS	NS	NS	W6979
*QSr.ufs-3D*	*wPt-732889 wPt-732908*	3D.2	LOD %VAR	NS	NS	NT	NT	3.75 8.33	NS	NS	NS	NS	NS	W6979

a *Only QTL with a LOD at or above significant threshold levels, as determined for each trait after 1000 permutations (P = 0.05), are shown. The LOD thresholds ranged from 3.0 to 11.5 in the W1406 x 37-07 population and from 3.0 to 3.2 in the W6979 x 37-07 population*.

b *Chromosome*.

c *Percentage phenotypic variance explained (r^2^)*.

d *NS, Not significant*.

e *Most significant marker varies at this QTL interval*.

f *NT, Not tested*.

**Figure 3:**
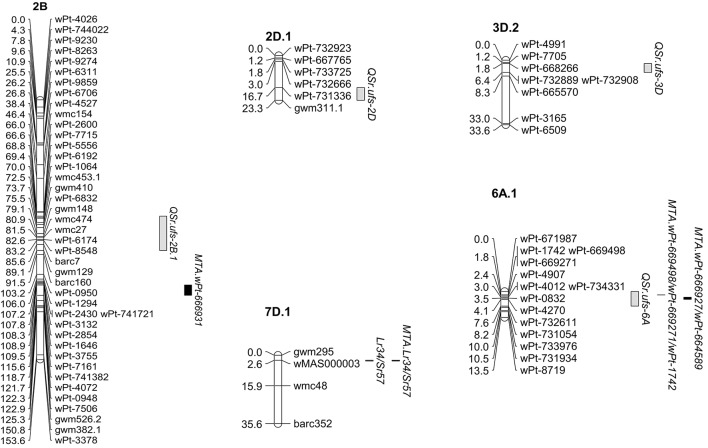
**Stem rust resistance detected in the wheat cross W6979 x 37-07**. The four QTL and *Lr34/Yr18/Sr57* found in the line W6979 are shown along with the MTA detected on the same linkage groups in the GWAS. The complete genetic map generated for the cross W6979 × 37-07 can be found in Supplementary Figure 8.

Five stem rust resistance QTL, all contributed by W6979, were identified in the W6979 × 37-07 cross (Table 6, Figure 3). Again the locus *Lr34/Yr18/Sr57* was found in W6979, but its effect was only detected with the SAI scores (*r*^2^ up to 11.02%). The biggest effect QTL was *QSr.ufs-6A* (*r*^2^ up to 16.32% for SAI and up to 17.02% for RT), located on chromosome 6AS this QTL was significant over all seasons and locations. Additional, minor QTL were identified on chromosomes 2BS (one SAI and one RT score), 2DL (one RT score) and 3DL (one SAI score). The QTL identified in the W1406 x 37-07 cross derived from parent 37-07, *QSr.ufs-4A* was not detected in the W6979 x 37-07 population.

## Discussion

The appearance of the *Pgt* Ug99 race lineage resulted in an international effort to identify new sources of stem rust resistance (http://wheatrust.cornell.edu). Many wheat germplasm collections have been screened at KALRO-Njoro and GWAS undertaken to identify effective stem rust resistance genes. While GWAS provide for greater resolution of gene position statistical power is significantly compromised by the genetic structure of the study population, with related genotypes resulting in the identification of false positive QTL (Korte and Farlow, 2013). Subsequent development of bi-parental mapping populations allows for the validation and further analysis of predicted QTL carrying regions. In this study we selected two lines to confirm, or not, the stem rust resistance QTL predicted through a GWAS.

The observed structure within the African wheat collection was primarily based on country of origin effects. The Sudanese wheat accessions presented as a genetically isolated group, with both haplotype analysis and GWAS failing to identify any known *Sr* genes in the wheat accessions from the Sudan. All the Sudanese wheat lines were prefixed “Jebel Mara.” Jebel Mara is an isolated volcanic massif in West Sudan with distinct agro-ecological growing conditions and this may explain the distinct genetic characteristics of wheat genotypes collected from this region. The wheat accessions from Ethiopia, Kenya and South Africa overlapped, but could be distinguished based on country of origin by the 2nd PC using both DArT and SSR/STS markers. The genetic overlap could be due to the intensive use of elite lines from international breeding efforts (e.g., CIMMYT line introductions, Supplementary Table 1) within national wheat breeding programs.

Pedigree information, seedling IT reactions to race PTKST and marker haplotype analyses supported the presence of the seedling resistance genes *Sr24, Sr31*, and *Sr36* in the African wheat collection (Supplementary Table 1). *Sr24* was common in the modern South African lines, the *Sr24* carrying cv. Agent having been commonly used in wheat breeding in South Africa (Pretorius et al., 2012). *Sr31* was observed in two Kenyan and one South African line, the Kenyan lines having the *Sr31* carrying cv. Kavkaz in their pedigree. The low frequency of *Sr31* in recent South African entries is due to the strict quality standards set by the South African baking and milling industry, and thus general avoidance of the 1B/1R translocation in cultivar development (Pretorius et al., 2012). *Sr36* was present in four lines from Kenya and one from South Africa. However, *Sr36* has now been defeated by the race TTTSK (Singh et al., 2015; Patpour et al., 2016). Haplotype and seedling IT scores were not compatible for *Sr33* and *Sr35*. *Sr33* and *Sr35* are derived from alien introgressions from *Aegilops tauschii* (Jones et al., 1991) and *Triticum monococcum* spp. (McIntosh et al., 1984), respectively and are therefore unlikely to be present in the collection. Furthermore, *Sr22, Sr26*, and *Sr39*, also introgressions from wild relatives, were not detected in the African wheat collection.

The main aim of this study was to identify novel field stem rust APR effective against the *Pgt* Ug99 lineage. Therefore, the African wheat collection was specifically screened to identify known sources of rust APR, including the rust APR loci *Lr34/Yr18/Sr57, Lr46/Yr29/Sr58* and *Sr2*. The marker *cssfr6* has proven diagnostic for the locus *Lr34/Yr18/Sr57* (Lagudah et al., 2009), and both the haplotype analysis and the GWAS supported the presence of this APR gene in lines from Kenya, Ethiopia, Tunisia, Morocco and recent South African breeding lines. The presence of *Lr34/Yr18/Sr57* in these lines is probably due to the inclusion of CIMMYT spring wheat materials in their development.

The haplotype analysis identified *Sr2* in 10 lines, eight from Kenya and two of the older lines from South Africa. This was fewer lines than expected given the inclusion of CIMMYT materials in the pedigrees of wheat accessions from Kenya and breeding lines from South Africa, although previous studies have suggested that *Sr2* is not common in current South African wheat germplasm (Pretorius et al., 2012). The GWAS identified stem rust APR MTA with marker alleles *wPt-741331, tPt-6487* and *wPt-4842* on chromosome 3BS. However, comparison with published maps (McNeil et al., 2008; Paux et al., 2008) suggests that these marker loci are proximal to *Sr2*. The inability to detect *Sr2* in the GWAS may be a consequence of the low frequency of this gene in the African wheat collection, a weakness of GWAS being its lack of power to detect rare alleles within a population.

While the haplotype analysis of the complex APR locus *Lr46/Yr29/Sr58* was inconclusive, the GWAS indicated the presence of this locus in many accessions, including the majority of the lines from Kenya, Ethiopia, Tunisia and South Africa, and all the lines from Morocco and Zambia. In the haplotype analysis *Lr46/Yr29/Sr58* was only called as present when all published linked markers had the same allele as the control genotype Pavon 76. However, in the GWAS *Lr46/Yr29/Sr58* was identified by only one gene marker; marker allele *ncw1-417bp* showing a significant MTA. Screening of wheat breeder's materials at CIMMYT (SD, unpublished data) and CenGen (RP, unpublished data) indicate limitations in the diagnostic potential of this marker, suggesting the presence of false positives in the GWAS analysis.

The GWAS identified 20 MTA within the African wheat collection associated with the SAI phenotypic data sets and 12 MTA with the RT data sets, with three MTA being common between SAI and RT, making a total of 29 stem rust resistance MTA. The wheat line W1406 carried the effective allele for 17 of these MTA, while W6979 contained 27 MTA. However, the only MTA confirmed by the QTL analysis of the bi-parental mapping populations were the locus *Lr34/Yr18/Sr57* on chromosome 7DS, being confirmed in both W1406 and W6979, the three W1406 MTA on chromosome 3BS, associated with DArT markers *wPt-741331, tPt-6487* and *wPt-4842* (Table 5), which lie close to the *QSr.ufs-3B* peak marker *cfa2226* (Table 6, Figure 2) and the five MTA in W6979 on chromosome 6AS (significant markers: *wPt-1742, wPt-669498* and *wPt-6669271;* Table 5) lying within the 3.9-6.8 cM region where the QTL *QSr.ufs-6A* peak marker *wPt-4270* is found (Table 6, Figure 3). Although *cfa2226* is closely linked to *gwm493*; previously reported to be linked to *Sr2* (Spielmeyer et al., 2003), W1406 tested negative for *Sr2* (3BS) both in the haplotype and GWAS analyses, and when screened with the additional *Sr2* associated marker *csSr2* (data not shown; Mago et al., 2011). Other stem rust resistance QTL have been reported in CIMMYT wheat materials in the region of *QSr.ufs-3B* (Crossa et al., 2007; Bhavani et al., 2011) and in the region of *QSr.ufs-6A* (Yu et al., 2011; Bansal et al., 2013). Given the origin and pedigree of W1406 and W6979 it is clear that CIMMYT wheat germplasm has been included in the development of these lines.

The W1406 stem rust resistance QTL *QSr.ufs-4D* was not detected in the GWAS, chromosome 4D being represented by only 4 DArT markers in the African wheat collection, none of which lay within the *QSr.ufs-4D* QTL region (Figure 2). Two stem rust resistance genes are reported for chromosome 4D, *Sr41* and the APR locus *Lr67/Yr46/Sr55. Sr41* is not effective against the Ug99 race lineage. However, *Lr67/Yr46/Sr55* has been reported to reduce stem rust infection by 41% in field trials in Mexico and a lower, but still significant reduction of 16% in the presence of race TTKST in Njoro, Kenya (Herrera-Foessel et al., 2014). The SSR marker *gwm192* maps 0.4 cM from *Lr67/Yr46/Sr55*, according to Herrera-Foessel et al. (2011). The *gwm192.2* allele, tightly linked with *QSr.ufs-4D* (Figure 2) therefore suggests that this QTL maybe the *Lr67/Yr46/Sr55* locus.

The W6979 QTL on chromosomes 2DL (*QSr.ufs-2D*) and 3DL (*QSr.ufs-3D*) were also not detected in the GWAS, however for these QTL there were DArT markers in common between the two studies. One DArT marker (*wPt-667765)* associated with *QSr.ufs-2D* and two DArT markers (*wPt-4991*; *wPt-668266*) associated with *QSr.ufs-3D* were present in the final selection of DArTs used in the GWAS. The failure to detect the MTA representing *QSr.ufs-2D* and *QSr.ufs-3D* in the GWAS may be due to the low level of genetic variance explained by each QTL and the variation seen in expression over growing seasons. To the best of our knowledge no stem rust resistance QTL effective against the Ug99 race lineage have been reported in the locations of the minor QTL *QSr.ufs-2D* and *QSr.ufs-3D* (Yu et al., 2011). However, it must be remembered that these minor QTL were not consistently detected across seasons and locations, and further characterization is required to confirm a true stem rust resistance gene at these QTL locations.

Both W1406 and W6979 carried a QTL located on chromosome 2BS. Peak marker loci: *barc7* in W1406 (Figure 2) and *gwm148* in W6979 (Figure 3), lie in close proximity to each other, suggesting that both lines may carry the same stem rust resistance QTL. However, the only stem rust resistance MTA found on chromosome 2B is with the 2B long arm marker *wPt-666931* (Table 5), which is approximately 40 cM away from these QTL peak markers. *QSr.ufs-2B* lies within a region of chromosome 2BS where several stem rust resistance genes/QTL have been found, including the genes *Sr36* and *Sr40* which are effective against the Ug99 race group (Yu et al., 2014). However, in seedling tests both W1406 and W6979 were susceptible to PTKST, and lines predicted to carry *Sr36* were removed from the GWAS. A stem rust resistance QTL effective against the Ug99 lineage, having been screened in Kenya in 2010 and 2011, was found in the same region in the Canadian wheat cv. AC Cadillac (Singh et al., 2013). However, the available pedigree information for W1404, W6979 and AC Cadillac does not support genetic similarity of these QTL.

The DArT Wheat Interpolated Maps v.4 were used to determine the relative position of markers showing significant MTA on the W1406 x 37-07 and W6979 x 37-07 genetic linkage maps. For the 2AS MTA (*wPt-743061* and *wPt-7187*) the corresponding region was not covered by a LG in the W1406 x 37-07 map. As the chromosome position of *wPt-667538* on 7A is unknown it was not possible to determine whether the genomic region containing this MTA was represented in the W1406 x 37-07 and W6979 x 37-07 7A LGs. All other MTA regions (Table 5) (W1406: 2BL, 2DS, 3BL, 5AS, 6AL, 6BS, 6DS, 7BL, and 7DS) and W6979: 1BS, 2AS, 2BL, 3BS, 3BL, 6AS, 6AL, 6DS, 7BL, and 7DS) were covered by a LG in the corresponding genetic maps, and therefore should have been detected as a QTL.

While all possible approaches to reduce false positive MTA were undertaken in this study the confounding effects of relatedness can result in the incorrect calling of MTA. Analysis of the population structure within the African wheat collection indicated that Bayesian clustering analysis had a greater probability of resulting in Type I errors. Therefore, only PCA was considered for the GWAS. The proportion of explained variance of these PCs was however low, with a maximum of 9.5%. Consequently we corrected for population stratification in the GWAS using different models, including population effects (Q2) and family structures (kinship matrices). More stringent criteria would also have resulted in fewer MTA being called in the GWAS, however it was noted that this would have resulted in *Lr34/Yr18/Sr57* going undetected. Similarly, a recent validation of previously reported MTA for agronomic traits, including grain yield, in barley validated only 33% of the MTAs (Lüders et al., 2016).

Trait-associated alleles present at low frequencies, and low marker coverage in the GWAS population can result in MTA going undetected. The extent of LD affects the number of markers required for association analyses. The LD value in the African wheat collection was comparable or lower than previous studies in wheat (Horvath et al., 2009; Chao et al., 2010), with markers on average being required to be within 5 cM of a stem rust resistance gene for a MTA to be significant. However, the low percentage of intra-chromosomal marker pairs (7.7%) in significant LD suggests that the marker coverage within the African wheat collection might be considered at the lower limit for a GWAS. In addition, some chromosomal regions; chromosomes 4D and 5D in particular, were not well covered with markers (Supplementary Table 4). Also, markers which are not in complete LD with stem rust resistance genes will lead to an underestimation of the explained genotypic variance of that gene.

Bi-parental mapping can resolve the effects of relatedness by breaking up the covariances between genotypes and phenotypes, enabling the detection of QTL associated with low frequency alleles (Myles et al., 2009). However, the relative low level of stem rust resistance phenotypic variance explained in both DH populations would suggest that other stem rust resistance QTL remained undetected. These could include the GWAS MTA that lay in genomic regions not represented by LGs in the bi-parental genetic maps. Genome-wide association and bi-parental mapping are therefore approaches to trait discovery that support and complement each other, and together support the effective utilization of the valuable collection of stem rust resistance identified within the African wheat collection.

## Author contributions

SD and RP performed the Genome-Wide Association Screen analysis of the African wheat collection. RP performed the QTL analyses of the double haploid mapping populations. ZP screened the African wheat collection and the mapping populations for stem rust resistance. DS screened the African wheat collection for stem rust resistance in the Kenyan field trials. LB planned and wrote the paper, assisted by SD, RP, and ZP. HS, EW, and CP screened the mapping populations with DNA markers, and CS also assisted with construction of the genetic maps. CB assisted with the scoring of stem rust resistance in Kenya and South Africa. DS, HS, EW, CS, and CB all contributed to the critical reading and editing of the manuscript. All authors have approved the manuscript submitted.

## Funding

The work in this manuscript was supported by the UK Biotechnology and Biological Sciences Research Council (BBSRC) special initiatives: Sustainable Agricultural Research for International Development (SARID) Project BB/F004125/1 and Sustainable Crop Production Research for International Development (SCPRID) Project BB/J011525/1.

### Conflict of interest statement

The authors declare that the research was conducted in the absence of any commercial or financial relationships that could be construed as a potential conflict of interest.
