# Supplementary material for: Stem Rust Resistance in a Geographically Diverse Collection of Spring Wheat Lines Collected from Across Africa
Source: Front Plant Sci. 2016 Jul 11;7:973. doi: 10.3389/fpls.2016.00973 (PMC4939729; doi:10.3389/fpls.2016.00973)
Supplement: Supplementary file 2 [file Table2.DOCX]

**Supplementary Table 2** SSR and STS markers associated with known stem rust genes

| Gene | Chr | Markers | Forward primer sequence (5’-3’) | Reverse primer sequence (5’-3’) | Control | Reference |
| --- | --- | --- | --- | --- | --- | --- |
| *Sr2* | 3BS | gwm533 | GTTGCTTTAGGGGAAAAGCC | AAGGCGAATCAAACTGAATA | Cranbrook,  Palmiet | Spielmeyer et al 2003 |
|  |  | gwm389 | ATCATGTCGATCTCCTTGACG | TGCCATGCACATTAGCAGAT |  | Spielmeyer et al 2003 |
|  |  | gwm493 | GGAACATCATTTCTGGACTTTG | TTCCCATAACTAAAACCGCG |  | Spielmeyer et al 2003 |
|  |  | stm559n | GGAGGGAAACTATCAAAATATGCTGGT | TGTGTGTGTGTGTGAGAGAGAG |  | Pretorius et al 2012 |
| *Sr22* | 7AL | cfa2019 | GACGAGCTAACTGCAGACCC | CTCAATCCTGATGCGGAGAT | Sr22B | Khan et al 2005 |
|  |  | cfa2123 | CGGTCTTTGTTTGCTCTAAACC | ACCGGCCATCTATGATGAAG |  | Yu et al 2010 |
|  |  | barc121 | ACTGATCAGCAATGTCAACTGAA | CCGGTGTCTTTCCTAACGCTATG |  | Yu et al 2010 |
|  |  | wmc633 | ACACCAGCGGGGATATTTGTTAC | GTGCACAAGACATGAGGTGGATT |  | Olson et al 2010 |
| *Sr24* | 3D | barc71 | GCGCTTGTTCCTCACCTGCTCATA | GCGTATATTCTCTCGTCTTCTTGTTGGTT | Palmiet | Mago et al 2005 |
|  |  | Sr24#50 | CCCAGCATCGGTGAAAGAA | ATGCGGAGCCTTCACATTTT |  | Pretorius et al 2012 |
| *Sr26* | 6A | Sr26#43 | AATCGTCCACATTGGCTTCT | CGCAACAAAATCATGCACTA | Avocet S | Mago et al 2005 |
| *Sr31* | 1B.1RS | iag95 | CTCTGTGGATAGTTACTTGATCGA | CCTAGAACATGCATGGCTGTTACA | Federation/Kavkaz | Mago et al 2002 |
| *Sr33* | 1D | barc152 | CTTCCTAAAATCGGGCAACCGCTTGTTG | GCGTAATGATGGGAGTGGCTATAGGGCAGTT | RL5405 | Sambasivam et al 2008 |
|  |  | cfd15 | CTCCCGTATTGAGCAGGAAG | GGCAGGTGTGGTGATGATCT |  | Sambasivam et al 2008 |
|  |  | wmc147^a^ | AGAACGAAAGAAGCGCGCTGAG | ATGTGTTTCTTATCCTGCGGGC |  | Somers et al 2004 |
| *Sr35* | 3A | gwm155 | CAATCATTTCCCCCTCCC | AATCATTGGAAATCCATATGCC | Mq12/5*G2191_Rsr35 | Babiker et al 2009 |
|  |  | wmc173^a^ | TGCAGTTGCGGATCCTTGA | TAACCAAGCAGCACGTATT |  | Somers et al 2004 |
| *Sr36* | 2B | stm773-2 | ATGGTTTGTTGTGTTGTGTGTAGG | AAACGCCCCAACCACCTCTCTC | SrTt1Sr36 | Tsilo et al 2008 |
|  |  | wmc477 | CGTCGAAAACCGTACACTCTCC | GCGAAACAGAATAGCCCTGATG |  | Tsilo et al 2008 |
| *Sr39/Lr35* | 2B | Sr39F2/R3 | AGAGAGAGTAGAAGAGCTGC | AGAGAGAGAGCATCCACC | RL6082 Sr39/Lr35 | Gold et al 1999 |
| *Lr34/Sr57* | 7DS | cssfr6 | CTGAGGCACTCTTTCCTGTACAAAG | GCATTCAATGAGCAATGGTTATC | Kariega | Lagudah et al 2009 |
|  |  |  |  |  |  | Prins et al 2011 |
| *Lr46/Sr58* | 1BL | barc80 | GCGAATTAGCATCTGCATCTGTTT GAG | CGGTCAACCAACTACTGCACAAC | Pavon F76 | MASwheat ^b^ |
|  |  | gwm140^a^ | ATGGAGATATTTGGCCTACAAC | CTTGACTTCAAGGCGTGACA |  | Somers et al 2004 |
|  |  | wmc44 | GGTCTTCTGGGCTTTGATCCTG | GTTGCTAGGGACCCGTAGTGG |  | Suenaga et al 2003 |
|  |  | ncw7 | Unpublished |  |  | Brown-Guedira^c^ |
|  |  | ncw1 | Unpublished |  |  | Brown-Guedira^c^ |
| ^a^Additional marker for region of interest as identified in wheat consensus map  ^b^http://maswheat.ucdavis.edu  ^c^Personal communication; not regarded as diagnostic marker | | | | | | |

**References**

Babiker E, Ibrahim AMH, Yen Y, Stein J. (2009) Identification of a microsatellite marker associated with stem rust resistance gene *Sr35* in wheat. Australian Journal of Crop Science 3(4):195-200.

Gold J, Harder D, Townley-Smith F, Aung T, Procunier J (1999) **Development of a molecular marker for rust resistance genes *Sr39* and *Lr35* in wheat breeding lines**. Plant Biotechnology 2(1) 1-5 Electronic Journal of Biotechnology ISSN 0717-3458

Khan RR, Bariana HS, Dholokia BB, Naik SV, Lagu MD, Rathjen AJ, Bhavani S and Gupta VS (2005) Molecular mapping of stem and leaf rust resistance in wheat. Theor Appl Genet 111:846-850. DOI 10.1007/s00122-005-0005-4.

Lagudah ES, Krattinger SG, Herrera-Foessel S, Singh RP, Huerta-Espino JH, Spielmeyer W, Brown-Guedira G, Selter LL, Keller B (2009) Gene-specific markers for the wheat gene *Lr34/Yr18/Pm38* which confers resistance to multiple fungal pathogens. Theor Appl Genet 119:889-898. DOI 10.1007/s00122-009-1097-z

Mago R, Bariana HS, Dundas IS, Spielmeyer W, Lawrence GJ, Pryor AJ and Ellis JG. (2005) Development of PCR markers for the selection of wheat stem rust resistance genes *Sr24* and *Sr26* in diverse wheat germplasm. Theor Appl Genet 111:496-504. DOI 10.1007/s00122-005-2039-z

Mago R, Spielmeyer W, Lawrence GJ, Lagudah ES, Ellis JG, Pryor A (2002) Identification and mapping of molecular markers linked to rust resistance genes located on chromosome 1RS of rye using wheat-rye translocation lines. Theor Appl Genet 104:1317–1324.

Olson EL, Brown-Guedira G, Marshall D, Stack E, Bowden RL, Jin Y, Rouse M, Pumphrey MO (2010) **Development of wheat lines having a small introgressed segment carrying stem rust resistance gene** Sr22**.** Crop Science 50:1823-1830.
[DOI:10.2135/cropsci2009.11.0652](http://dx.doi.org/10.2135/cropsci2009.11.0652).

Pretorius ZA, Jin Y, Bender CM, Herselman L. & Prins R. 2012. Seedling resistance to stem rust race Ug99 and marker analysis for *Sr2*, *Sr24* and *Sr31* in South African wheat cultivars and lines. Euphytica 186 (1):15-23 DOI 10.1007/s10681-011-0476-0

Prins R, Pretorius ZA, Bender CM, Lehemensiek A (2011) QTL mapping of stripe, leaf and stem rust resistance genes in a Kariega X Avocet S doubled haploid wheat population. Mol Breeding 27:259-270. DOI 10.1007/s11032-010-9428-y.

Sambasivam PK, Bansal UK, Hayden MJ, Dvorak J, Lagudah ES, Bariana HS (2008) Identification of markers linked with stem rust resistance genes Sr33 and Sr45. In: Appels R, Eastwood R, Lagudah E, Langridge P, Mackay M, McIntyre L, Sharp P (eds). Proceedings of 11th International Wheat Genetics Symposium, Sydney University Press, Sydney, Australia pp: 351-353.

Somers DJ, Isaac P, Edwards K. (2004) A high-density microsatellite consensus map for bread wheat (*Triticum aestivum* L). Theor Appl Genet 109:1105-1114.

Spielmeyer W, Sharp PJ and Lagudah ES. Identification and validation of markers linked to broad-spectrum stem rust resistance gene *Sr2* in wheat (*Triticum aestivum* L.). 2003. Crop Science 43: 333-336.

Suenaga K, Singh RP, Huerta-Espino J, William HM (2003) Microsatellite Markers for Genes *Lr34/Yr18* and Other Quantitative Trait Loci for Leaf Rust and Stripe Rust Resistance in Bread Wheat. Phytopathology, 93(7):881-890.

Tsilo TJ, Jin Y, Anderson JA (2008) Diagnostic microsatellite markers for the detection of stem rust resistance gene *Sr36* in diverse genetic backgrounds of wheat. Crop Sci 48: 253-261.

Yu L-X, Liu S, Anderson JA, Singh RP, Jin Y, Dubcovsky J, Brown-Guidera G, Bhavani S, Morgounov A, He Z, Huerta-Espino J, Sorrells ME (2010) Haplotype diversity of stem rust resistance loci in uncharacterized wheat lines. Mol. Breeding 26: 667-680.
