# Supplementary material for: Stem Rust Resistance in a Geographically Diverse Collection of Spring Wheat Lines Collected from Across Africa
Source: Front Plant Sci. 2016 Jul 11;7:973. doi: 10.3389/fpls.2016.00973 (PMC4939729; doi:10.3389/fpls.2016.00973)
Supplement: Supplementary file 3 [file Table3.DOCX]

**Supplementary Table 3** Analyses of variance of SAI in the DH populations W1406 x 37-07 and W6979 x 37-07 over field trial seasons and locations

**W1406 x 37-07 DH population ANOVA**

| Source of variation | SS | Df | MS | F ratio | p-value |
| --- | --- | --- | --- | --- | --- |
| Lines | 208538.4 | 180 | 1158.5 | 5.723 | <0.001 |
| Trials | 530.2 | 4 | 132.5 | 0.655 | 0.624 |
| Error | 145760.0 | 720 | 202.4 |  |  |
| Total | 354828.5 | 904 |  |  |  |

| Trials | Count | Sum | Average | Variance |
| --- | --- | --- | --- | --- |
| Greytown 2012 | 181 | 3511.0 | 19.40 | 354.36 |
| Greytown 2014 | 181 | 3369.0 | 18.61 | 445.73 |
| Greytown 2015 | 181 | 3786.0 | 20.92 | 311.69 |
| Makhathini 2013 | 181 | 3635.8 | 20.09 | 477.08 |
| Makhathini 2014 | 181 | 3614.5 | 19.97 | 379.47 |

**W6979 x 37-07 DH population ANOVA**

| Source of variation | SS | df | MS | F ratio | p-value |
| --- | --- | --- | --- | --- | --- |
| Lines | 158619.4 | 175 | 906.4 | 5.452 | <0.001 |
| Trials | 13463.5 | 3 | 4487.8 | 26.996 | <0.001 |
| Error | 87277.2 | 525 | 166.2 |  |  |
| Total | 259360.1 | 703 |  |  |  |

| Trials | Count | Sum | Average | Variance |
| --- | --- | --- | --- | --- |
| Greytown 2012 | 176 | 2145.0 | 12.19 | 188.24 |
| Greytown 2014 | 176 | 4168.0 | 23.68 | 646.18 |
| Greytown 2015 | 176 | 3569.0 | 20.28 | 322.25 |
| Makhathini 2014 | 176 | 2765.0 | 15.71 | 248.45 |
