# Supplementary material for: Stem Rust Resistance in a Geographically Diverse Collection of Spring Wheat Lines Collected from Across Africa
Source: Front Plant Sci. 2016 Jul 11;7:973. doi: 10.3389/fpls.2016.00973 (PMC4939729; doi:10.3389/fpls.2016.00973)
Supplement: Supplementary file 4 [file Table4.DOCX]

| **Supplementary Table 4.** Genome coverage of 2185 DArT markers in the African wheat collection. DArT markers were assigned a chromosomal location based on the Wheat Interpolated Maps v4 (Diversity Arrays Technology Pty Ltd, personal communication) | | | | | |
| --- | --- | --- | --- | --- | --- |
| Chromosome | No of markers | Genetic distance  (cM) | ^a^Marker coverage | Min and max distance between markers | ^b^PIC |
| Unmapped | 481 | - | - | - | 0.255 |
| Mapped | 1704 | 2625.5 | 1.54 | 0, 109.9 | 0.299 |
| 1A | 140 | 134.9 | 0.96 | 0, 19.7 | 0.322 |
| 1B | 108 | 109.0 | 1.01 | 0, 8.5 | 0.292 |
| 1D | 40 | 130.8 | 3.27 | 0, 48.8 | 0.331 |
| 2A | 64 | 115.8 | 1.81 | 0, 14.2 | 0.291 |
| 2B | 144 | 129.9 | 0.9 | 0, 7.1 | 0.303 |
| 2D | 69 | 98.1 | 1.42 | 0, 25.2 | 0.317 |
| 3A | 71 | 178.2 | 2.51 | 0, 30.0 | 0.296 |
| 3B | 175 | 125.5 | 0.72 | 0, 16.4 | 0.293 |
| 3D | 60 | 155.4 | 2.59 | 0, 81.5 | 0.303 |
| 4A | 83 | 107.6 | 1.3 | 0, 15.5 | 0.299 |
| 4B | 37 | 106.3 | 2.87 | 0, 20.2 | 0.277 |
| 4D | 4 | 66.6 | 16.65 | 0, 66.6 | 0.298 |
| 5A | 28 | 100.4 | 3.59 | 0, 51.3 | 0.288 |
| 5B | 103 | 165.9 | 1.61 | 0, 16.1 | 0.302 |
| 5D | 4 | 25.8 | 6.44 | 0, 21.4 | 0.213 |
| 6A | 154 | 108.8 | 0.71 | 0, 20.3 | 0.301 |
| 6B | 130 | 130 | 1.00 | 0, 13.1 | 0.293 |
| 6D | 24 | 132.3 | 5.51 | 0, 60.8 | 0.280 |
| 7A | 92 | 170.1 | 1.85 | 0, 20.6 | 0.313 |
| 7B | 78 | 159.2 | 2.04 | 0, 27.6 | 0.295 |
| 7D | 96 | 174.9 | 1.82 | 0, 109.9 | 0.274 |
| Total | 2185 | - | - | - | 0.290 |

^a^Marker coverage represents the genetic distance (cM) covered by markers on a chromosome divided by the number of markers on that chromosome.

^b^The Polymorphism Information Content (PIC)
