# Supplementary material for: Stem Rust Resistance in a Geographically Diverse Collection of Spring Wheat Lines Collected from Across Africa
Source: Front Plant Sci. 2016 Jul 11;7:973. doi: 10.3389/fpls.2016.00973 (PMC4939729; doi:10.3389/fpls.2016.00973)
Supplement: Supplementary file 5 [file DataSheet1.DOCX]

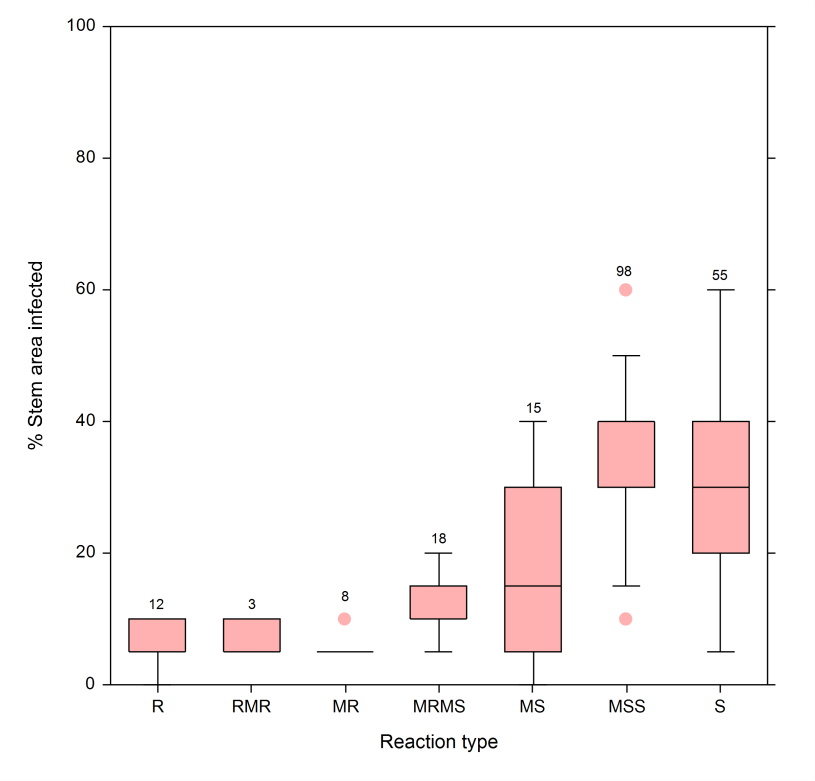

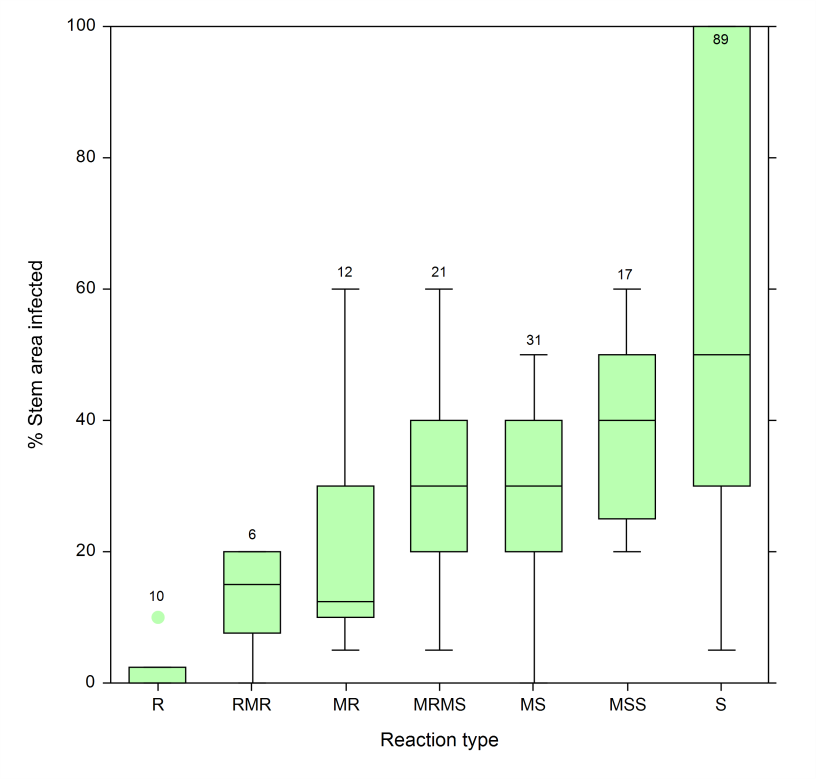

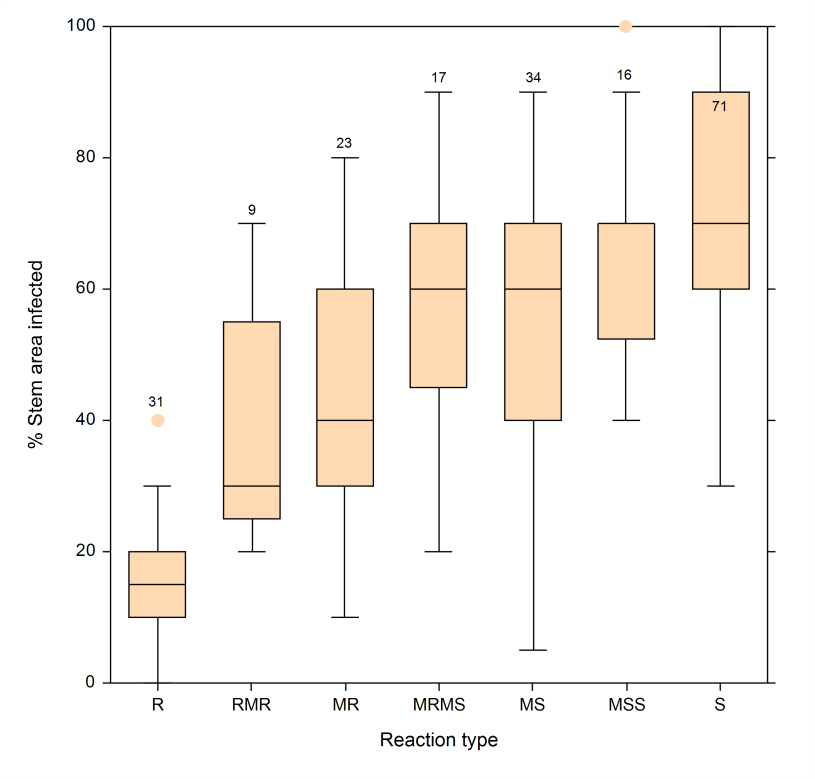


**Supplementary Figure 1** Box plots of stem rust infection (SAI) scores among wheat genotypes scored at (**A**) Njoro, Kenya in March 2009 (SAI1 scores: 209 genotypes), (**B**) Njoro, Kenya in September 2009 (SAI2 scores: 201 genotypes) and (**C**) Greytown, South Africa in November 2011 (SAI3 scores: 186 genotypes). The host reaction types are measured as resistant (R), moderately resistant (MR), moderately susceptible (MS) to susceptible (S). The coloured boxes represent the inter-quartile range (IQR; mid 50% data values) of stem rust severity observed for each reaction type. Horizontal lines within each box denote the median. The whisker boundaries were determined by multiplying the IQR by a factor of 1.5. Outliers are indicated by dots.

C

A

B
