# Supplementary material for: Stem Rust Resistance in a Geographically Diverse Collection of Spring Wheat Lines Collected from Across Africa
Source: Front Plant Sci. 2016 Jul 11;7:973. doi: 10.3389/fpls.2016.00973 (PMC4939729; doi:10.3389/fpls.2016.00973)
Supplement: Supplementary file 6 [file DataSheet2.DOCX]

**Supplementary Figure 2** Analysis of population structure within the African wheat collection calculated by Bayesian clustering analysis using the software Structure v2.2; **(A**) ΔK plot and (**B**) proportion of explained variance of the first ten principal coordinates

B

A
