# Supplementary material for: Stem Rust Resistance in a Geographically Diverse Collection of Spring Wheat Lines Collected from Across Africa
Source: Front Plant Sci. 2016 Jul 11;7:973. doi: 10.3389/fpls.2016.00973 (PMC4939729; doi:10.3389/fpls.2016.00973)
Supplement: Supplementary file 7 [file DataSheet3.DOCX]

**Supplementary Figure 3** Intra-chromosomal linkage disequilibrium (LD) decay of marker pairs over all chromosomes as a function of genetic distance. The Loess fitting curve illustrates the LD decay while the horizontal line indicates an *r^2^* threshold of 0.1.


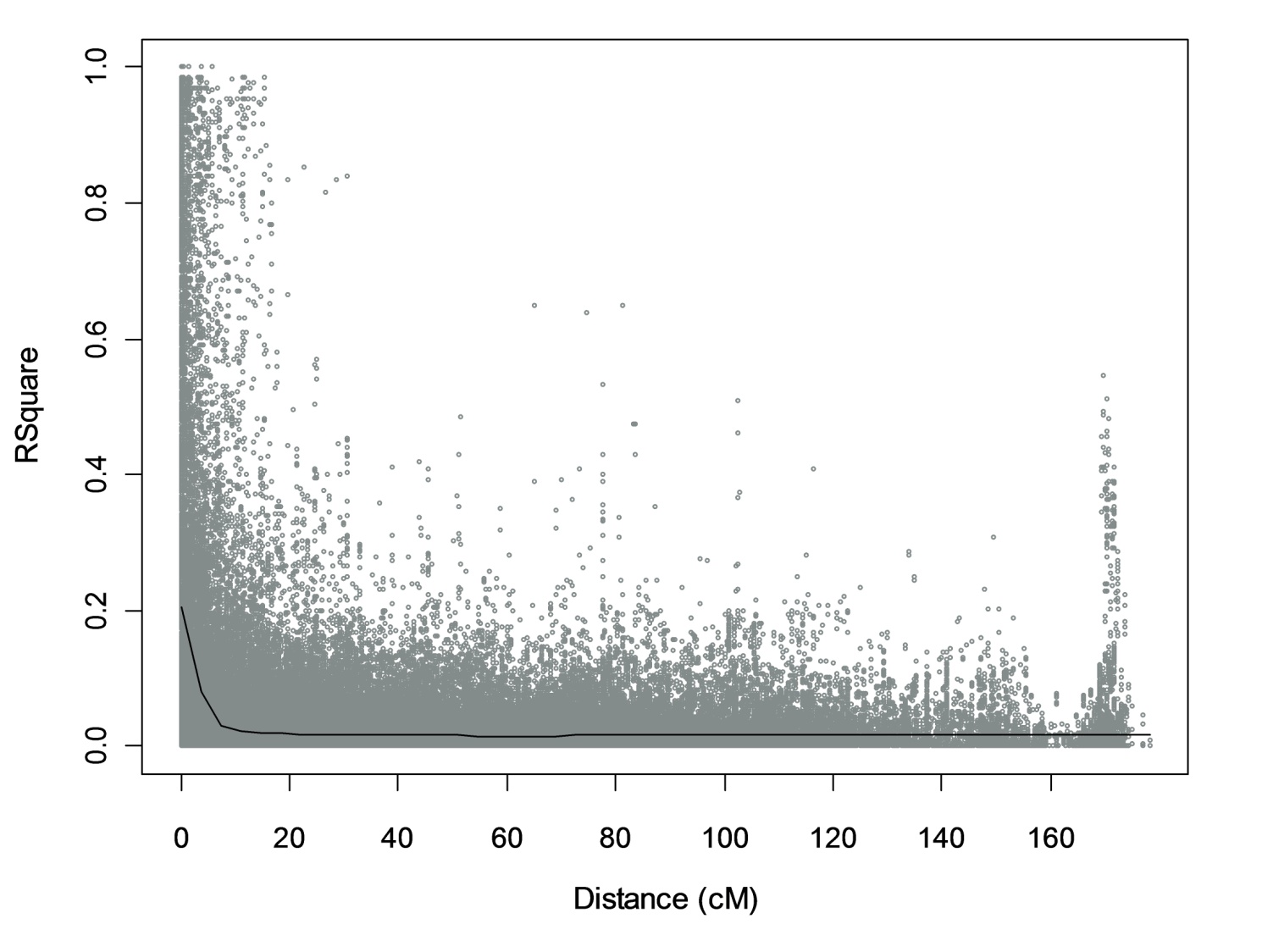


Unmapped markers
