# Supplementary material for: Stem Rust Resistance in a Geographically Diverse Collection of Spring Wheat Lines Collected from Across Africa
Source: Front Plant Sci. 2016 Jul 11;7:973. doi: 10.3389/fpls.2016.00973 (PMC4939729; doi:10.3389/fpls.2016.00973)
Supplement: Supplementary file 8 [file DataSheet4.DOCX]

**Supplementary Figure 4** Comparison of cumulative and observed *p-values* for each phenotypic data set calculated using 2185 DArT markers.
