# Supplementary material for: Stem Rust Resistance in a Geographically Diverse Collection of Spring Wheat Lines Collected from Across Africa
Source: Front Plant Sci. 2016 Jul 11;7:973. doi: 10.3389/fpls.2016.00973 (PMC4939729; doi:10.3389/fpls.2016.00973)
Supplement: Supplementary file 9 [file DataSheet5.DOCX]

**Supplementary Figure 5** GWAS analysis of the region on chromosome 2BS where *Sr* seeding resistance was identified using the Q2 model for statistical correction of population structure in the African wheat collection. Pair wise linkage disequilibrium (*r^2^*) between the most significant marker (*stm7732N-153bp*) and neighboring markers is presented by a black line.

*Significance threshold*
