# Supplementary material for: Stem Rust Resistance in a Geographically Diverse Collection of Spring Wheat Lines Collected from Across Africa
Source: Front Plant Sci. 2016 Jul 11;7:973. doi: 10.3389/fpls.2016.00973 (PMC4939729; doi:10.3389/fpls.2016.00973)
Supplement: Supplementary file 10 [file DataSheet6.DOCX]

**Supplementary Figure 6A** Distribution of stem rust Stem Area Infected (SAI) in the DH population of cross W1406 x 37-07 to *Puccinia graminis* f. sp. *tritici* race PTKST. Stem rust resistance was assessed in field trials at Pannar Research Station, Greytown in 2012 (Nov), 2014 (Nov) and 2015 (Oct), and at Makhathini Research Station, Jozini, South Africa in 2013 (Jul) and 2014 (Aug). These field trials were scored once within a season at each location.

**Supplementary Figure 6B** Distribution of stem rust Stem Area Infected (SAI) in the DH population of cross W6979 x 37-07 to *Puccinia graminis* f. sp. *tritici* race PTKST. Stem rust resistance was assessed in field trials at Pannar Research Station, Greytown in 2012 (Nov), 2014 (Nov) and 2015 (Oct), and at Makhathini Research Station, Jozini, South Africa in 2014 (Aug). These field trials were scored once within a season at each location.

**Supplementary Figure 6C** Distribution of stem rust host Reaction Types (RT) in the DH population of cross W1406 x 37-07 to *Puccinia graminis* f. sp. *tritici* race PTKST. Stem rust resistance was assessed in field trials at Pannar Research Station, Greytown in 2012 (Nov), 2014 (Nov) and 2015 (Oct), and at Makhathini Research Station, Jozini, South Africa in 2013 (Jul) and 2014 (Aug). These field trials were scored once within a season at each location.

**Supplementary Figure 6D** Distribution of stem rust host Reaction Types (RT) in the DH population of cross W6979 x 37-07 to *Puccinia graminis* f. sp. *tritici* race PTKST. Stem rust resistance was assessed in field trials at Pannar Research Station, Greytown in 2012 (Nov), 2014 (Nov) and 2015 (Oct), and at Makhathini Research Station, Jozini, South Africa in 2014 (Aug). These field trials were scored once within a season at each location.
