# Supplementary figures and images for: Stem Rust Resistance in a Geographically Diverse Collection of Spring Wheat Lines Collected from Across Africa
Source: Front Plant Sci. 2016 Jul 11;7:973. doi: 10.3389/fpls.2016.00973 (PMC4939729; doi:10.3389/fpls.2016.00973)

# 1A

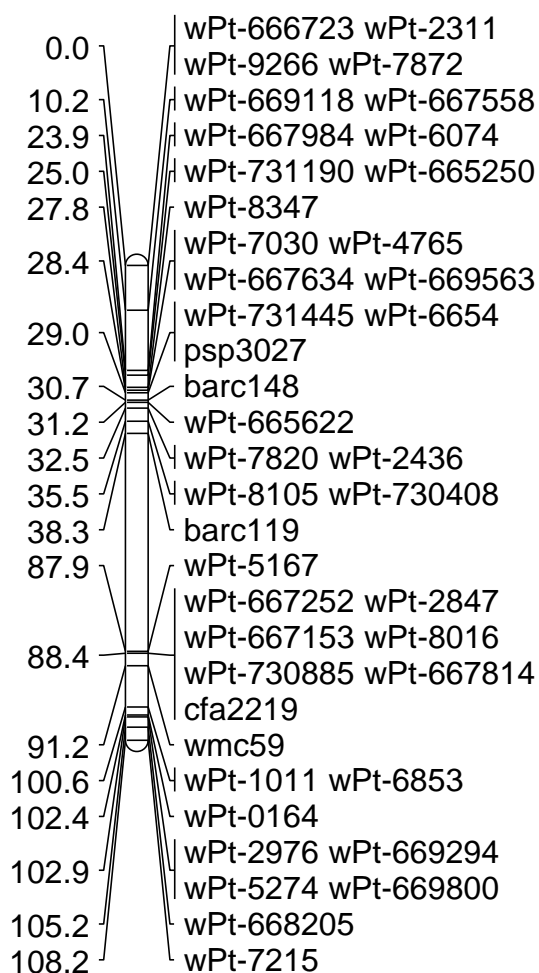

# 1B

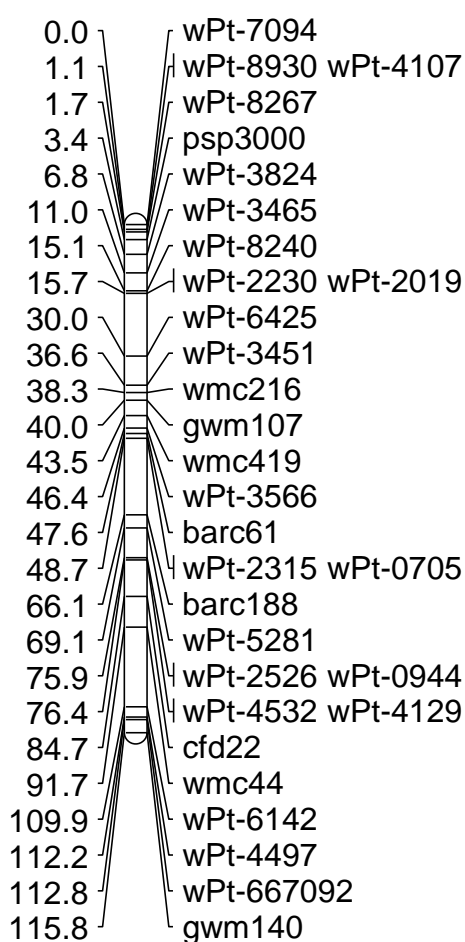

# 1D.1

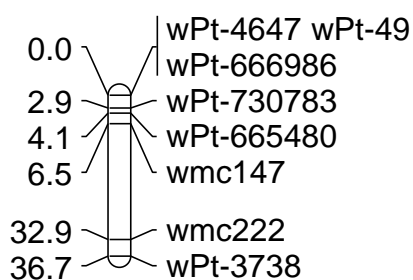

# 1D.2

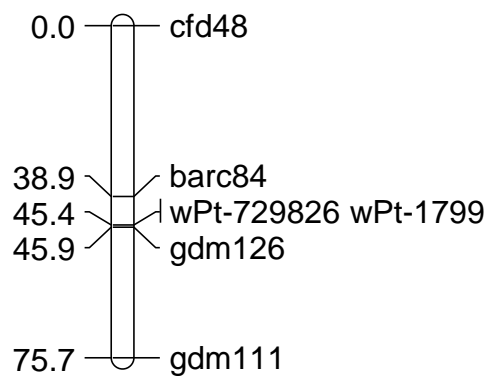

## 2A

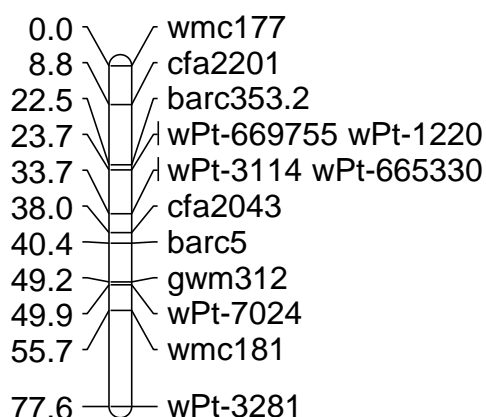

## 2B

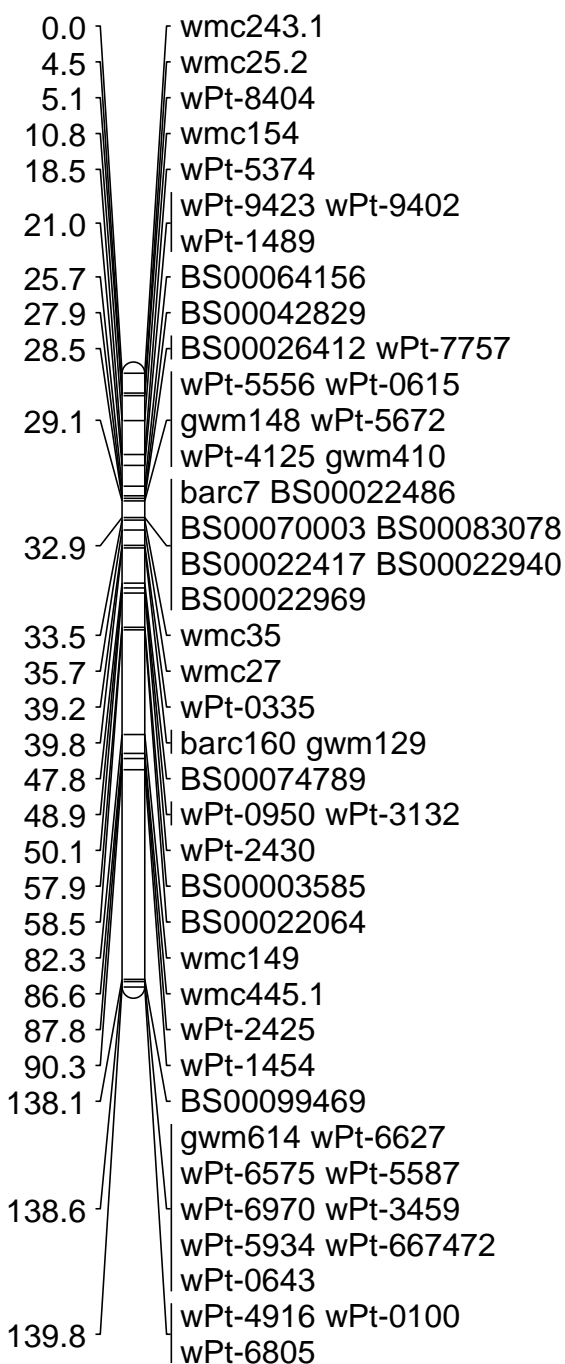

## 2D.1

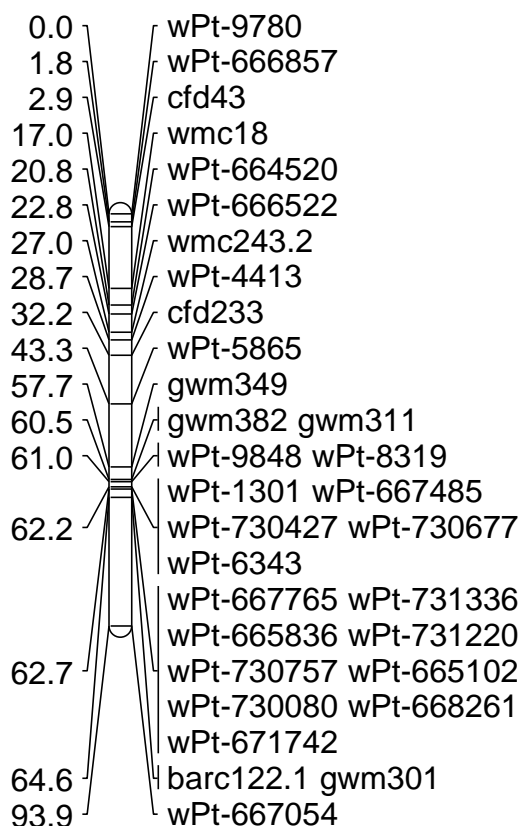

## 2D.2

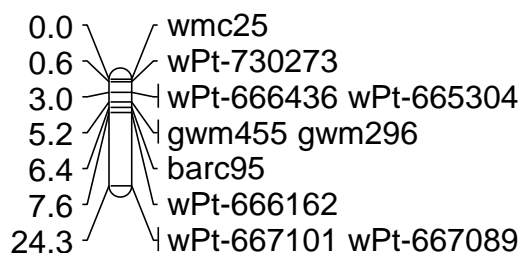

### 3A.1

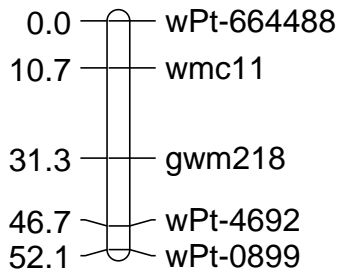

### 3A.2

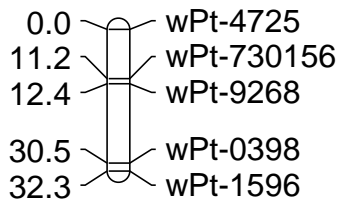

### 3B

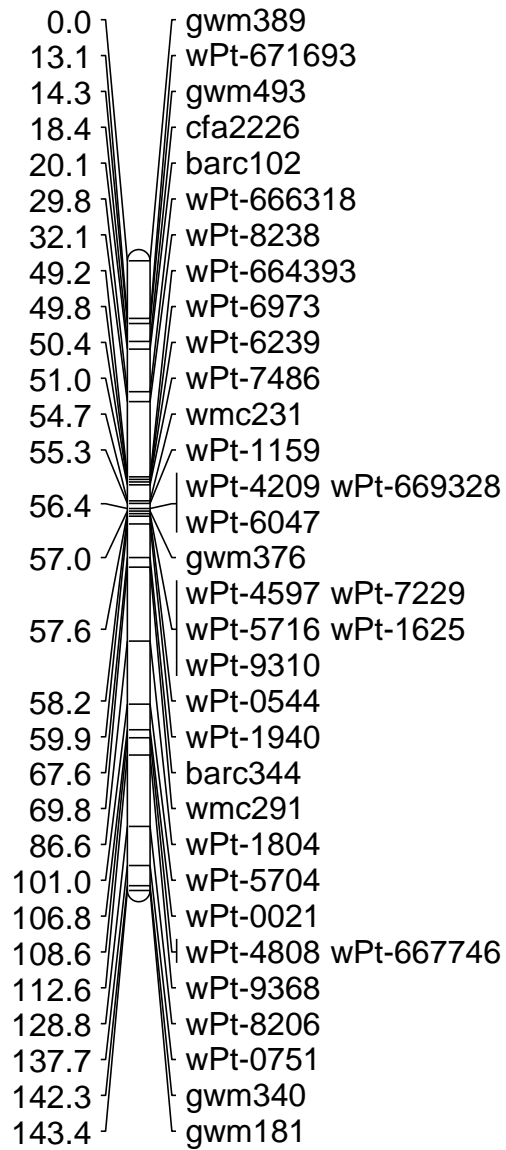

### 3D

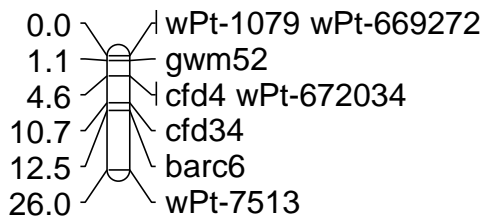

#### 4A

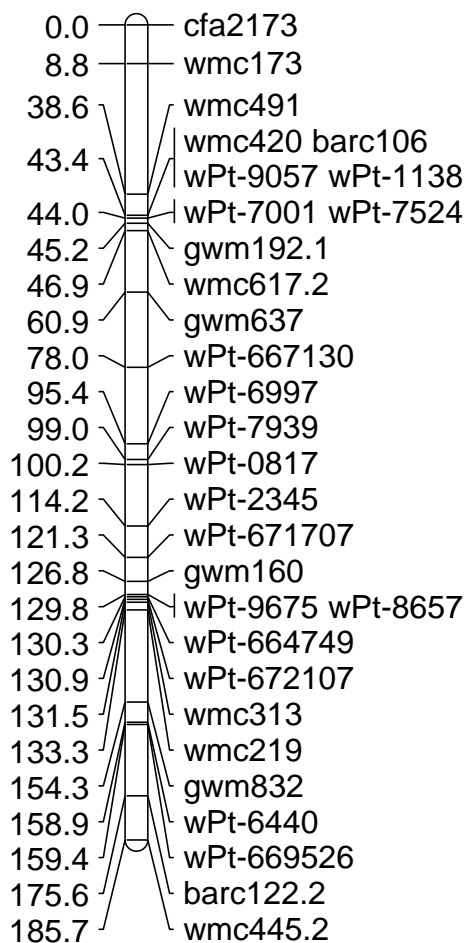

#### 4B

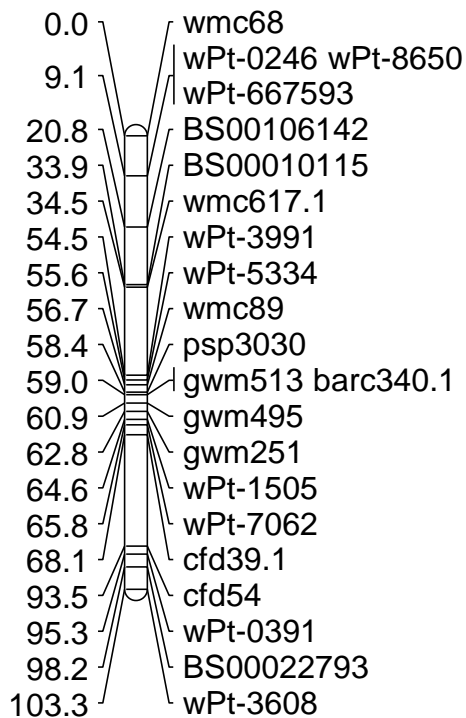

#### 4D.1

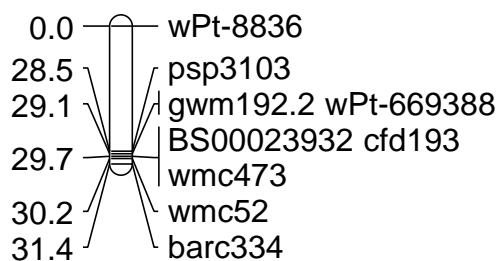

#### 4D.2

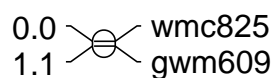

### 5A.1

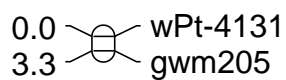

### 5A.2

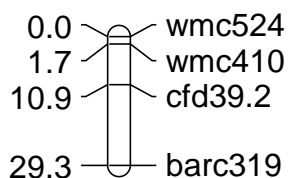

### 5D.1

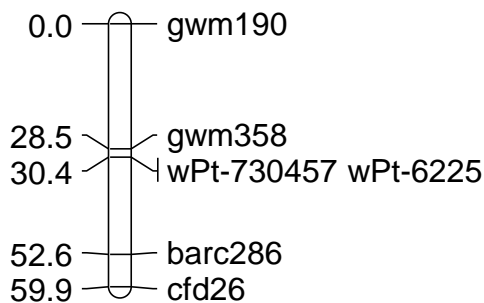

### 5D.2

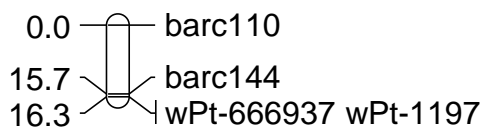

### 5B.1

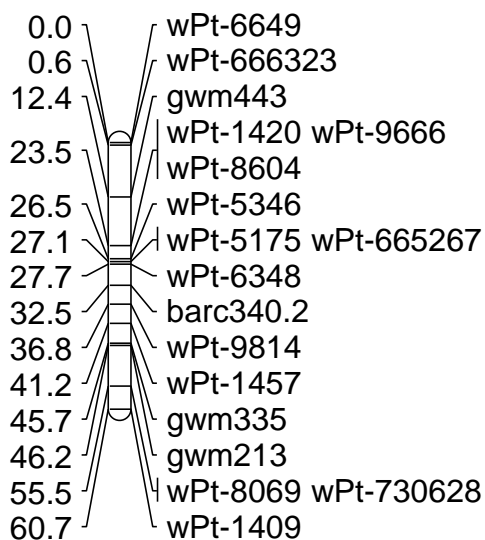

### 5B.2

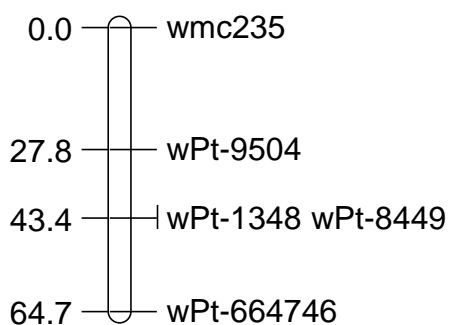

## 6A

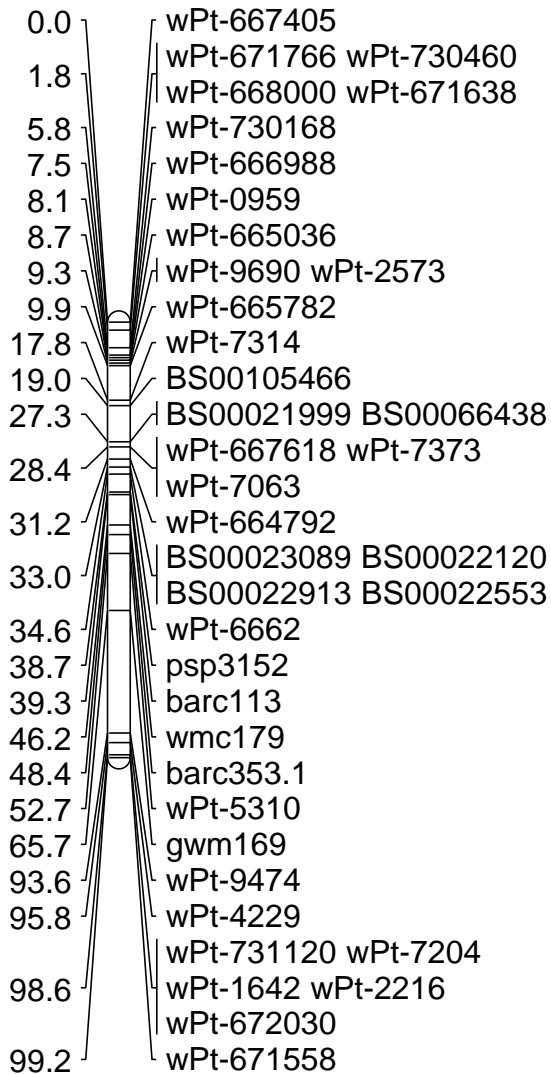

## 6B

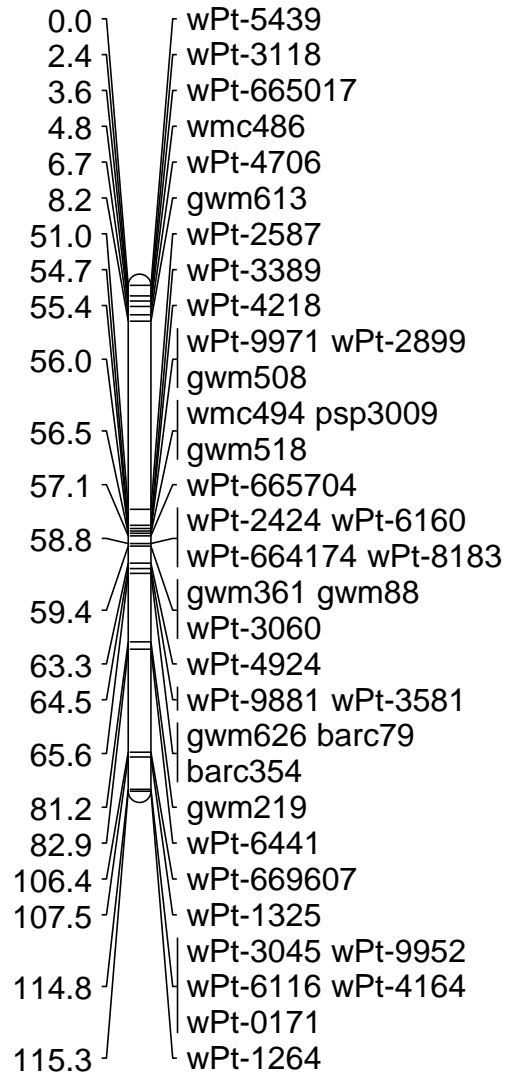

## 6D.1

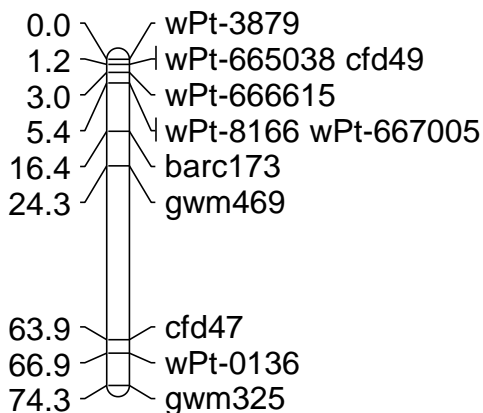

## 6D.2

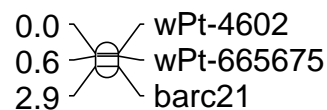

## 7A

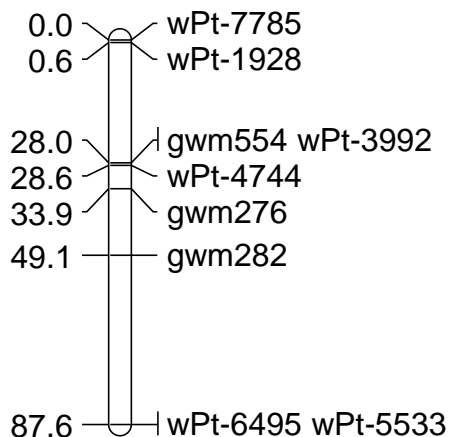

## 7B

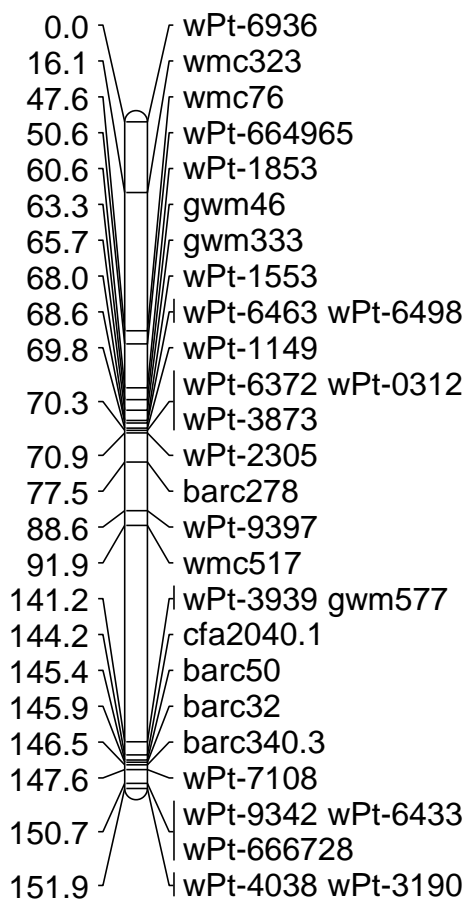

## 7D

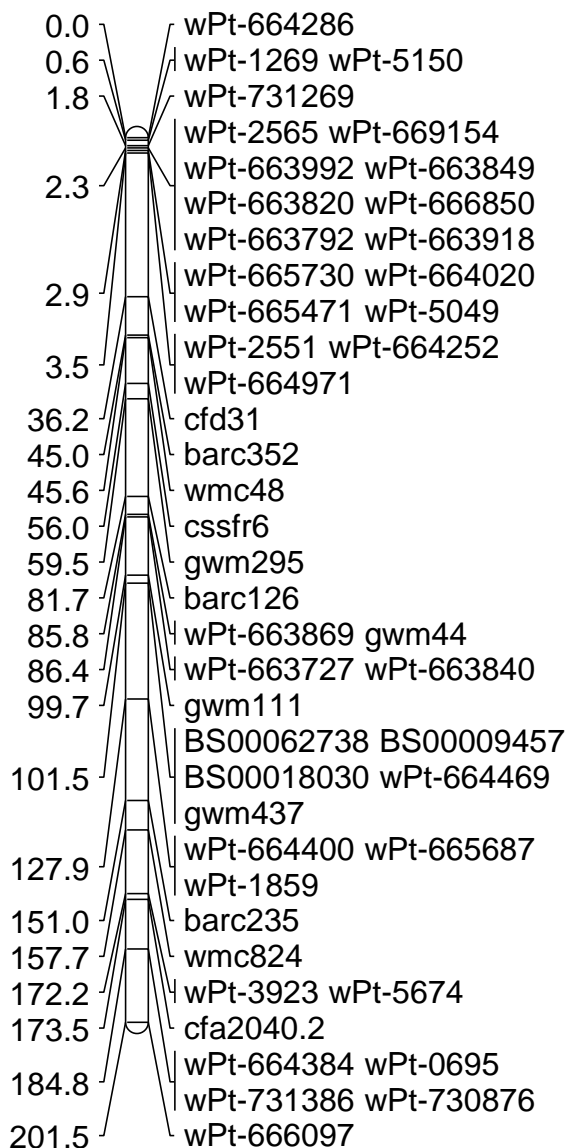

Supplement: Supplementary file 11 [file DataSheet7.PDF]

# 1A

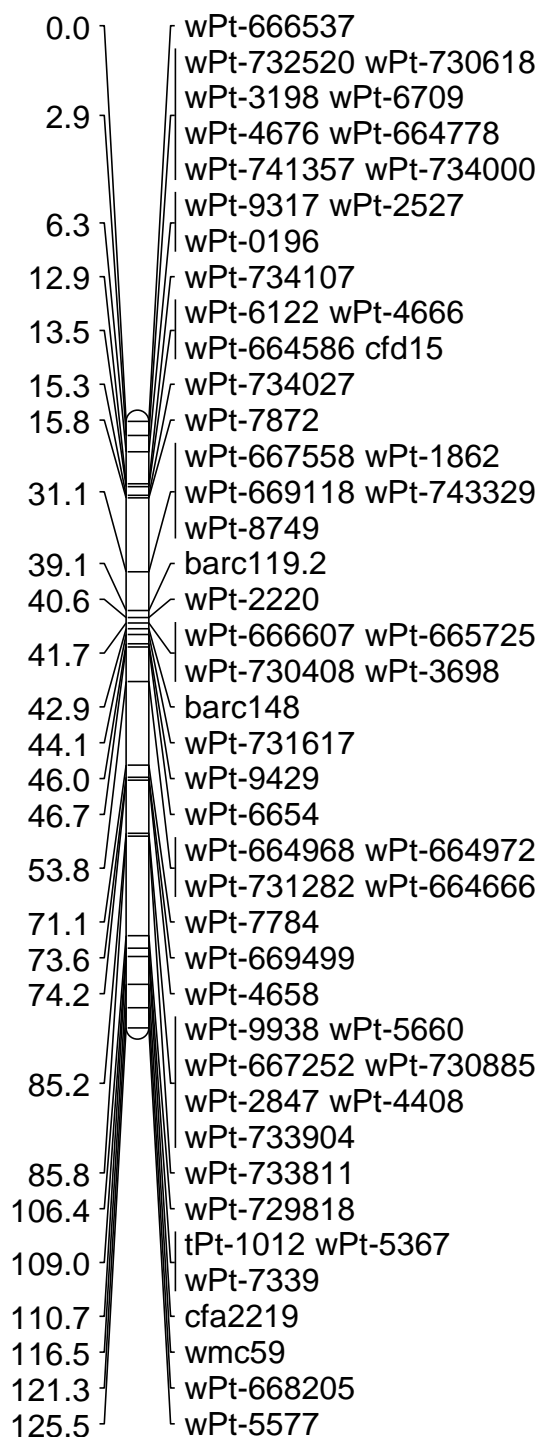

# 1B.1

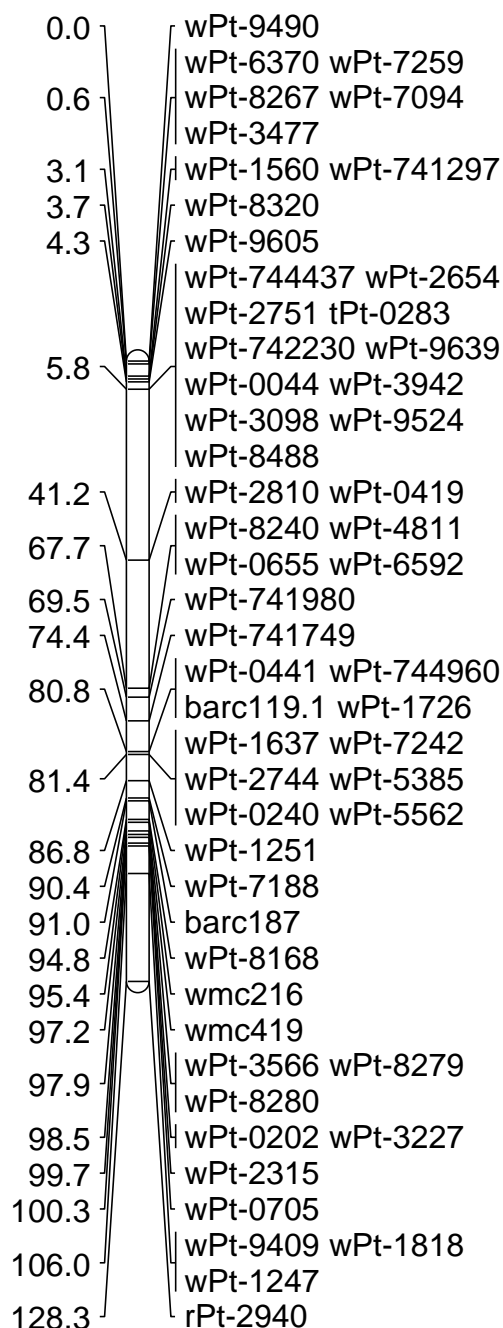

## 1B.2

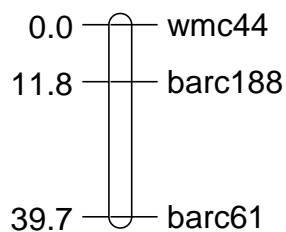

## 1D

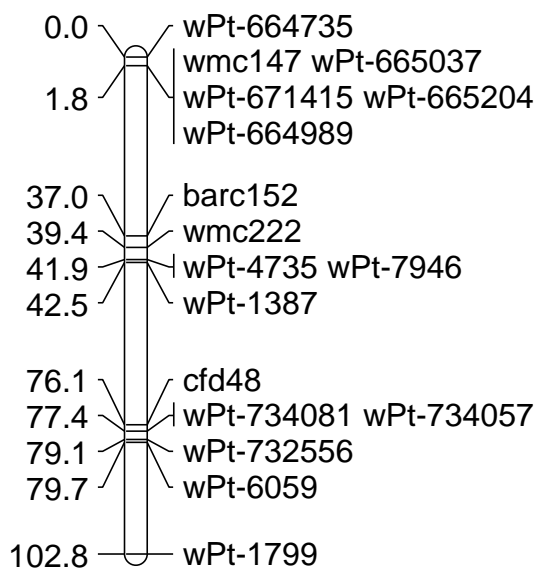

## 2A

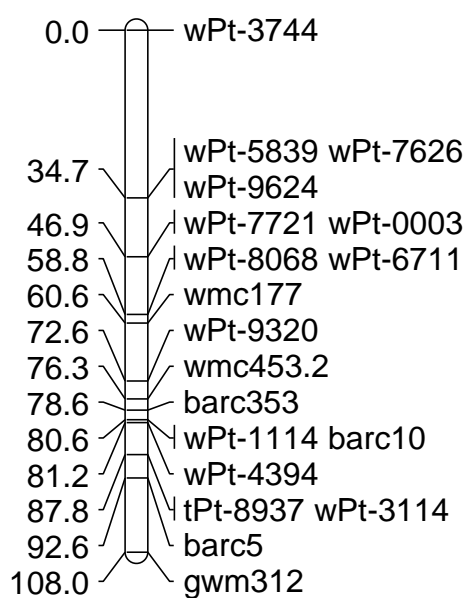

## 2B

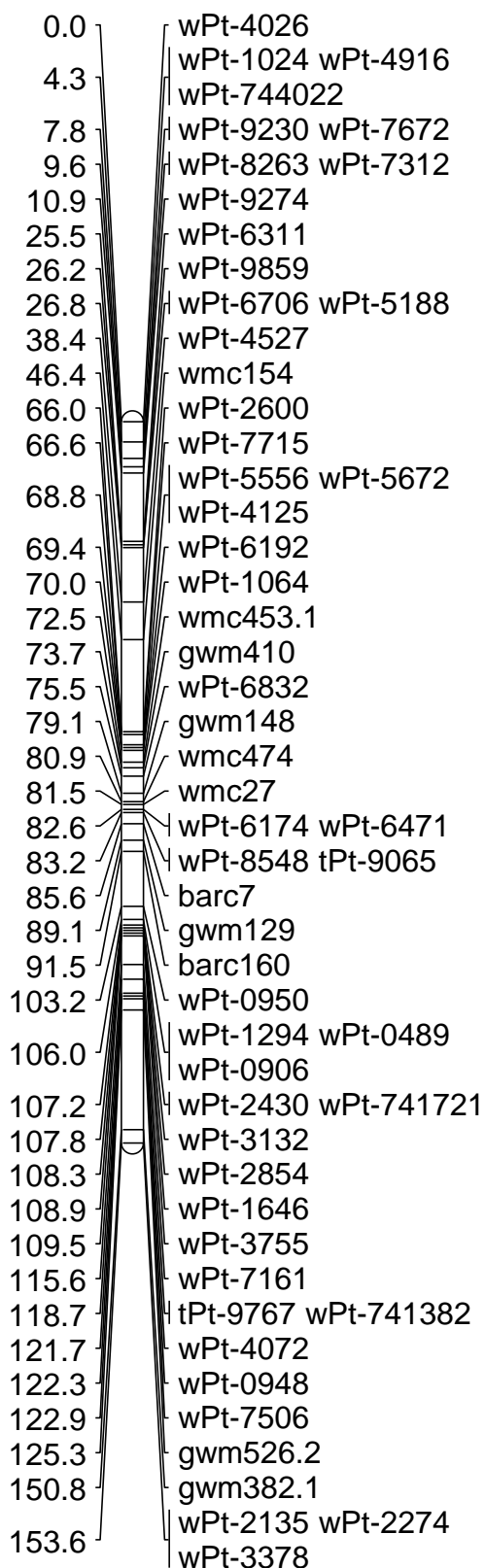

## 2D.1

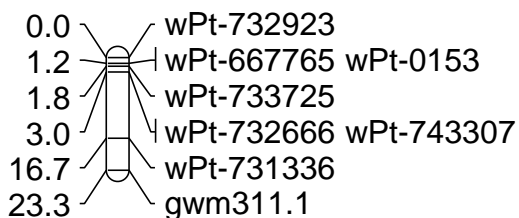

## 2D.2

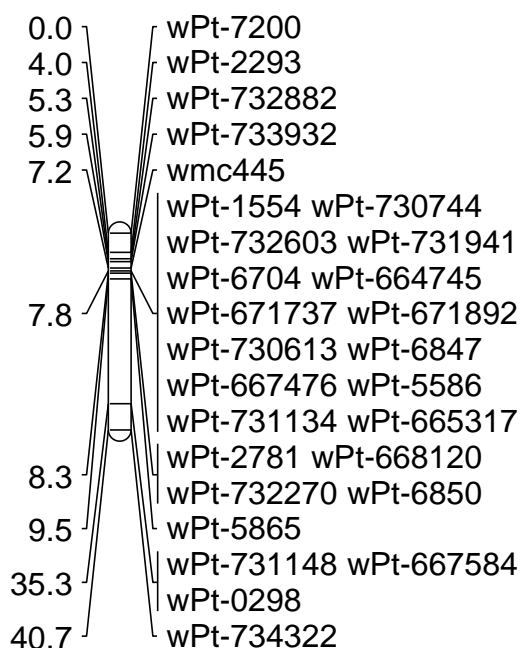

## 2D.3

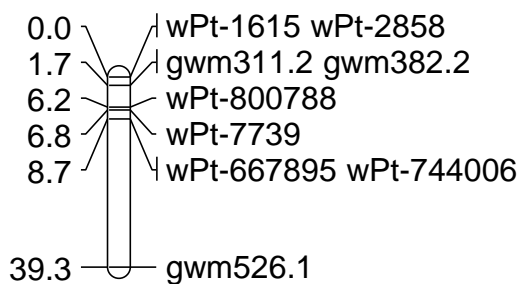

### 3A

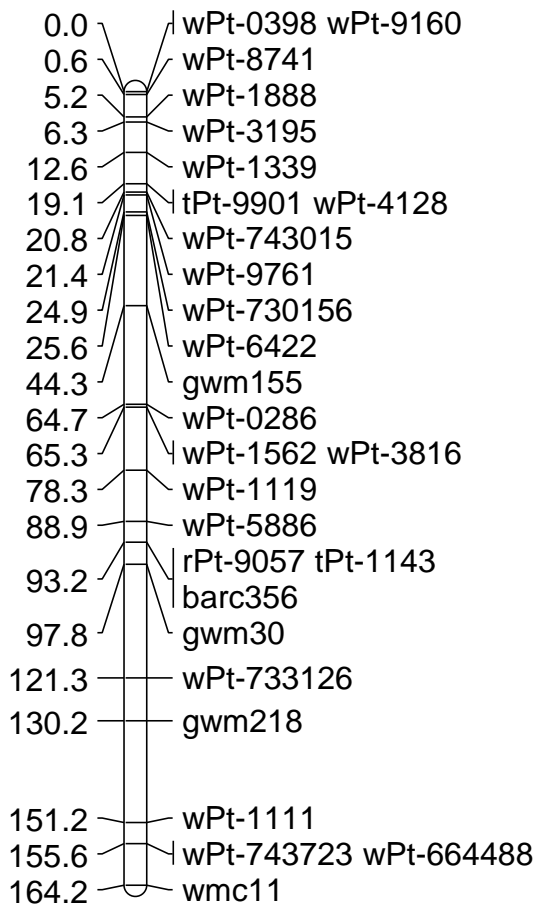

### 3B.1

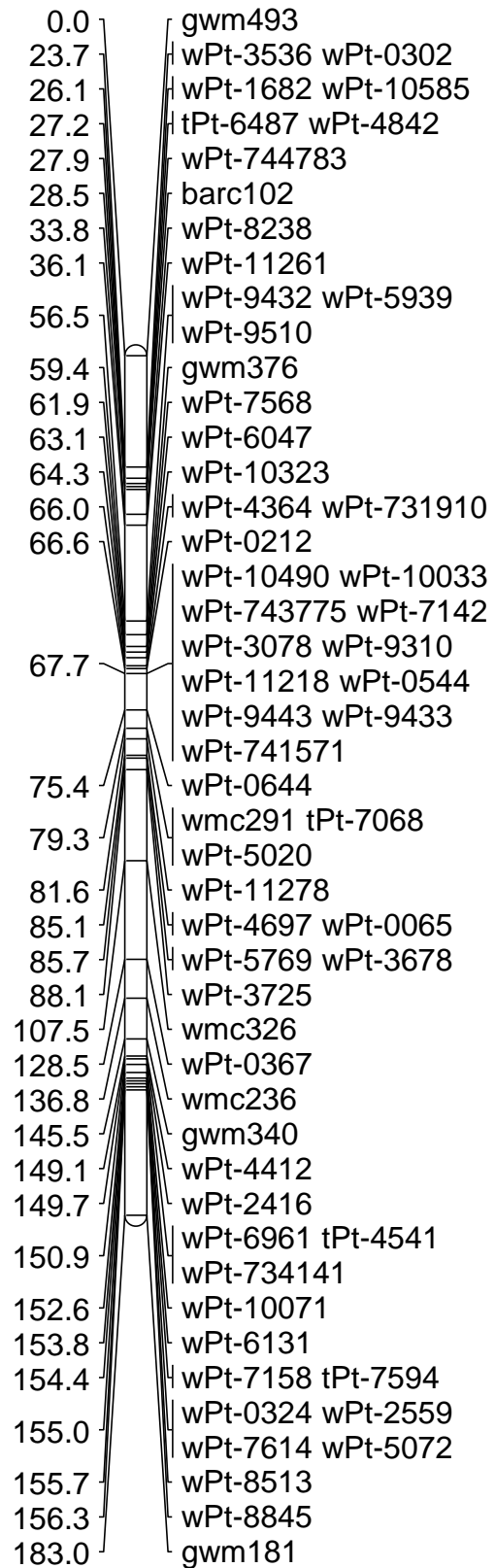

### 3B.2

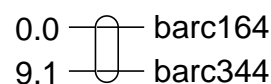

### 3D.1

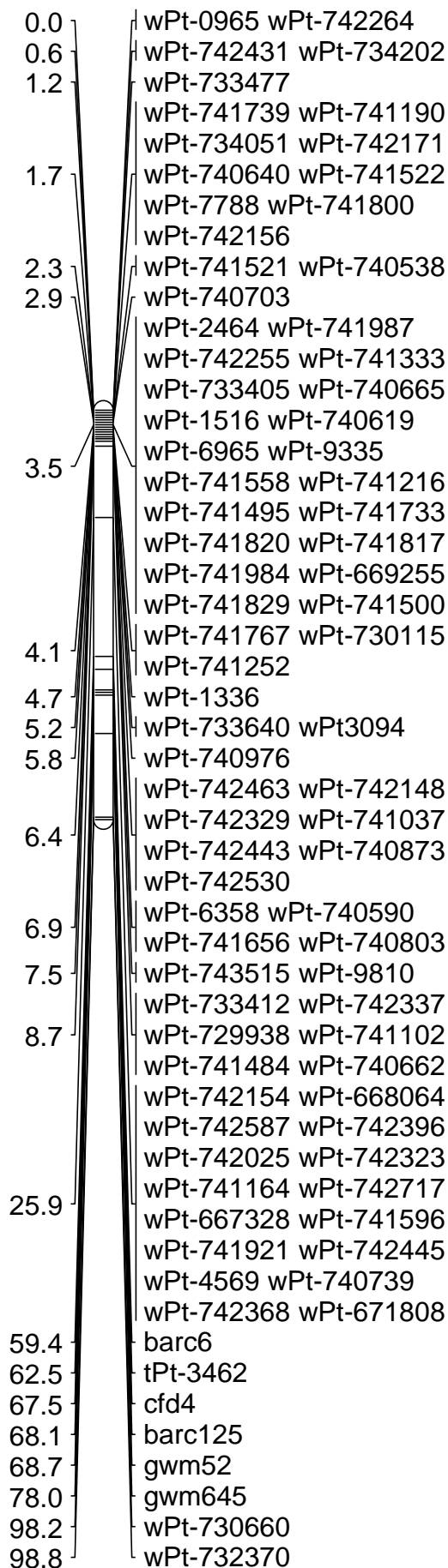

### 3D.2

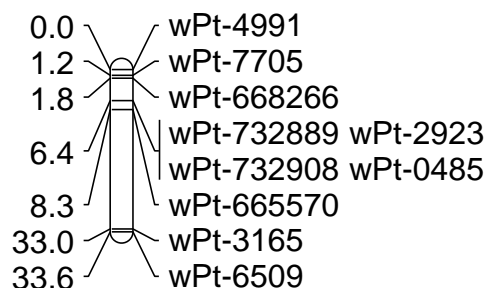

### 4A.1

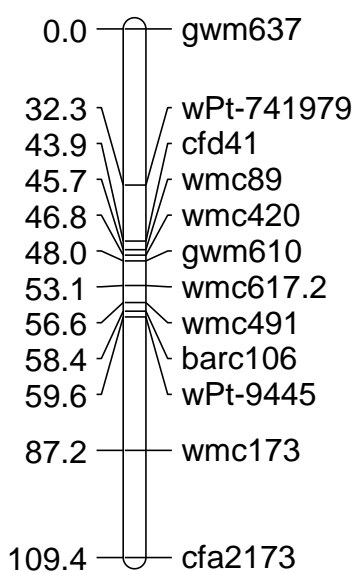

### 4A.2

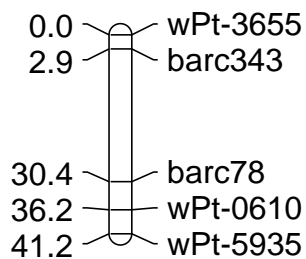

### 4B

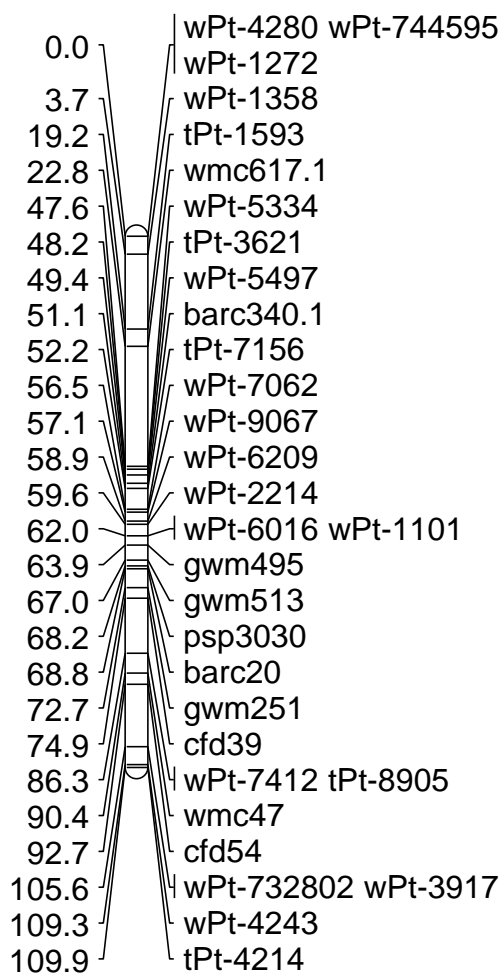

### 4D.1

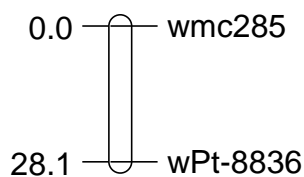

### 4D.2

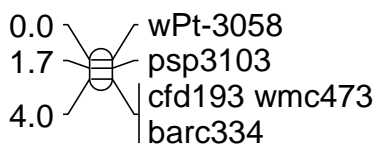

### 4D.3

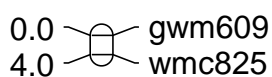

### 5A.1

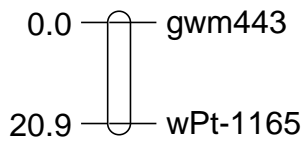

### 5A.2

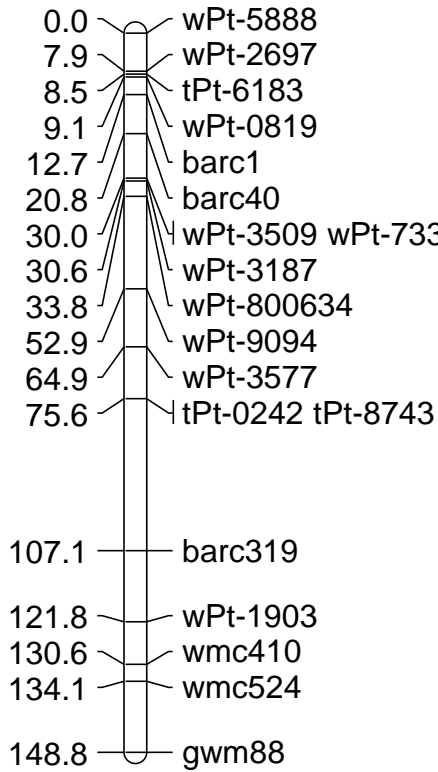

### 5B

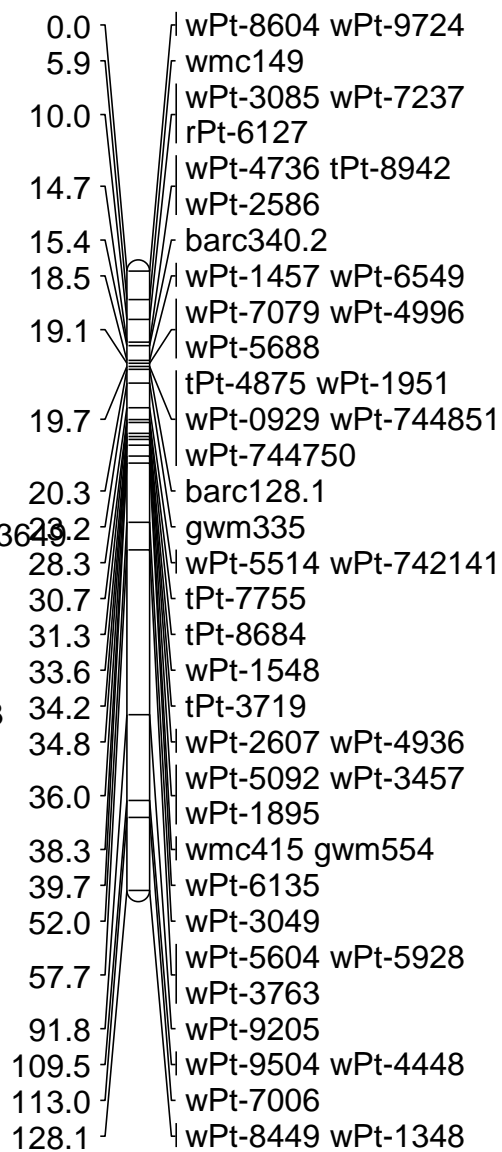

### 5D

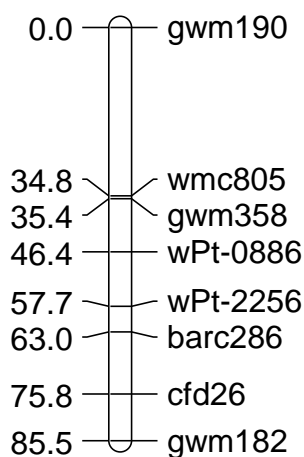

### 6A.1

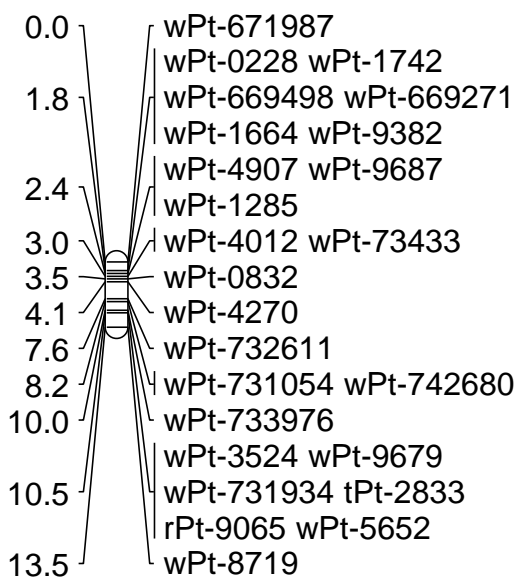

### 6A.2

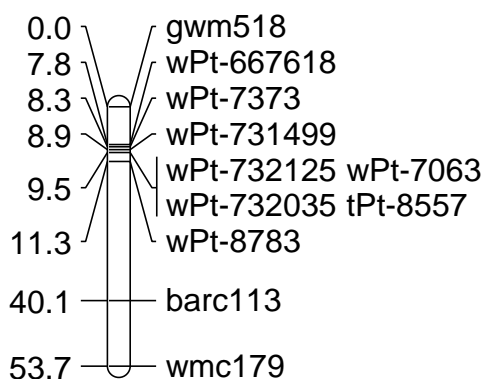

### 6A.3

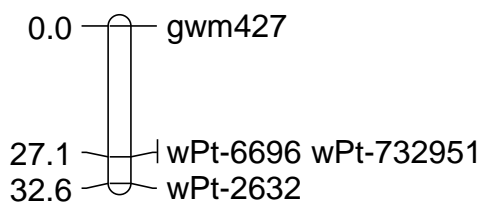

### 6B.1

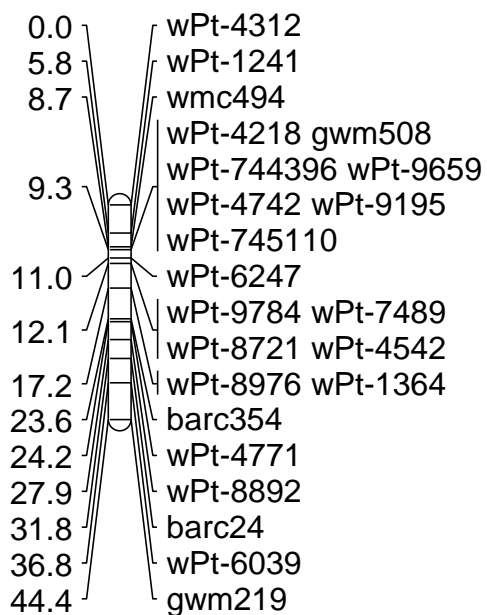

### 6B.2

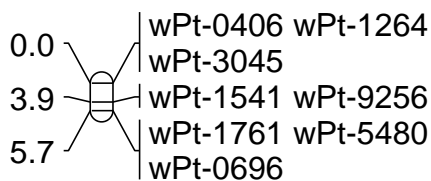

### 6D

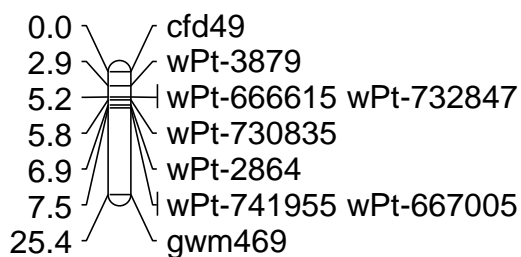

## 7A.1

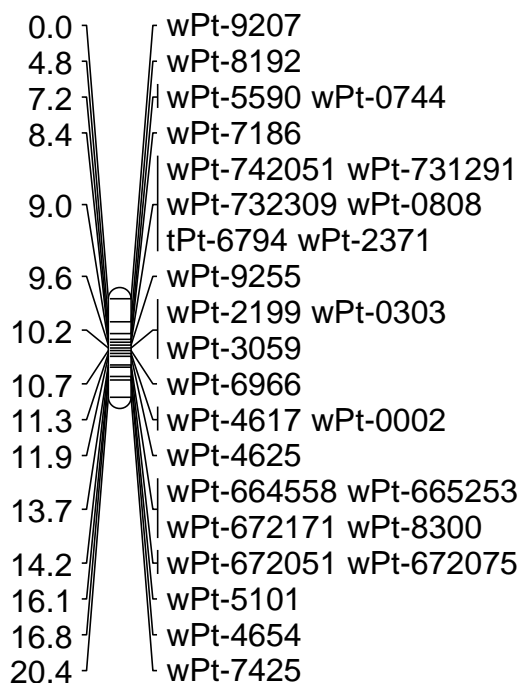

## 7B.1

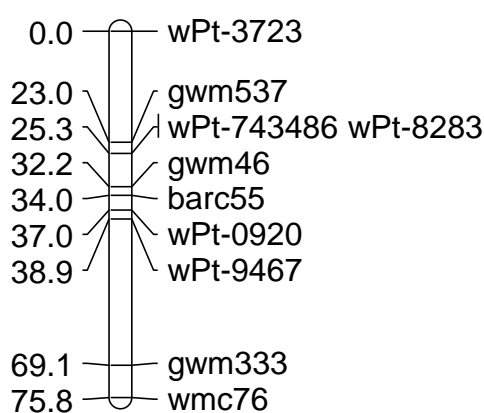

## 7A.2

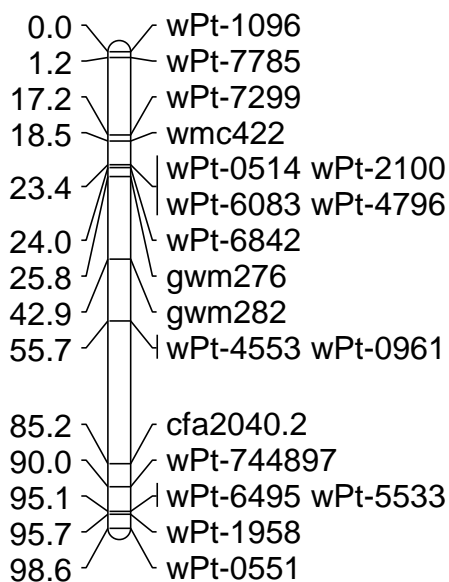

## 7B.2

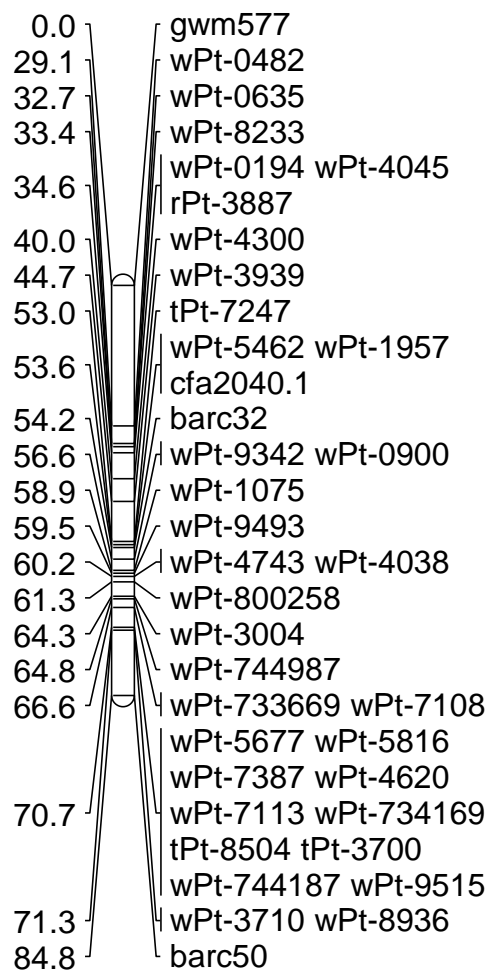

### 7D.1

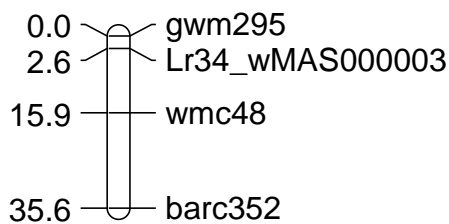

### 7D.2

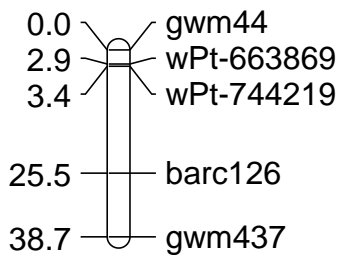

### 7D.3

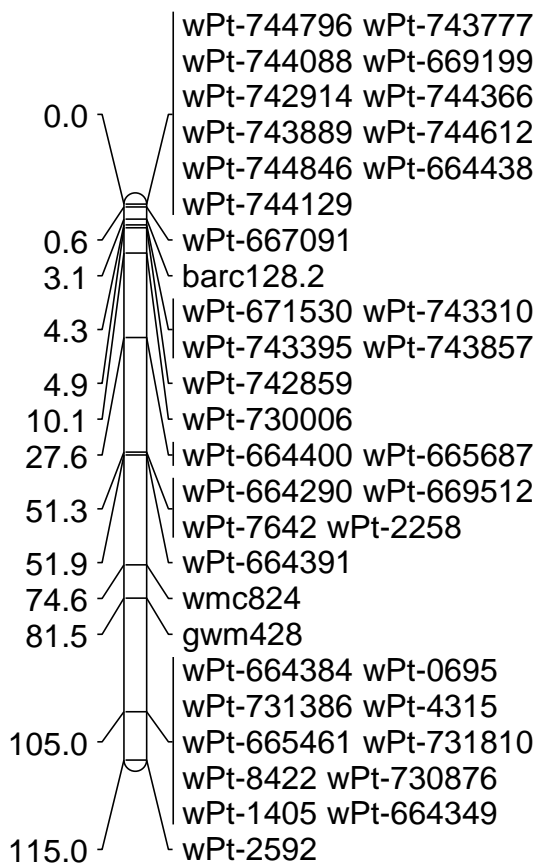

### 7D.4

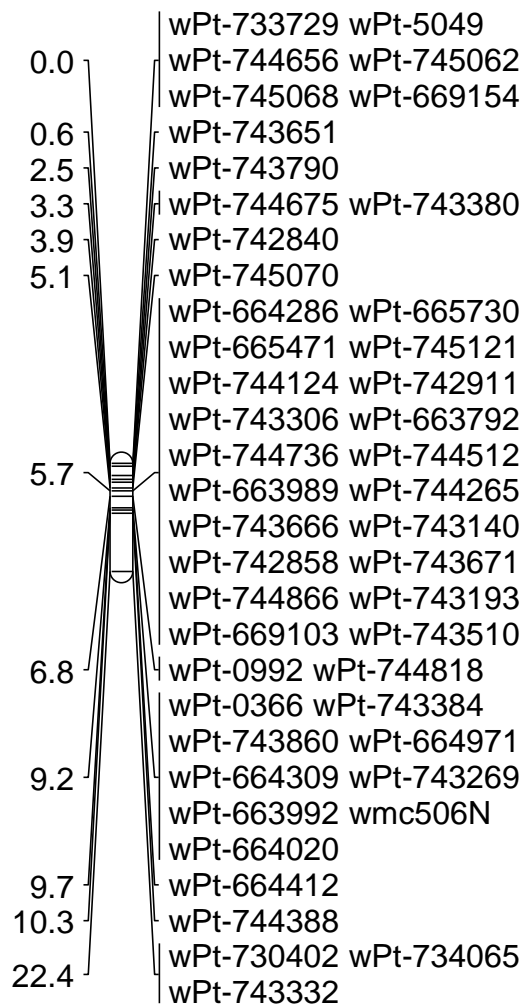

## UN1

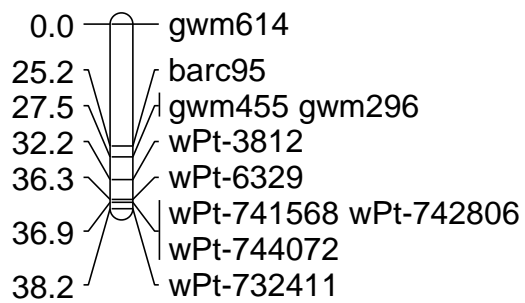

## UN2

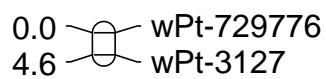

## UN3

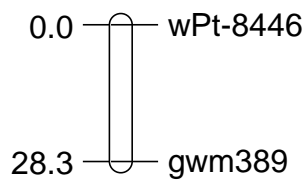

Supplement: Supplementary file 12 [file DataSheet8.PDF]
